# An Empirical Investigation of the Relationship Between Spirituality, Work Culture, and Burnout: The Need for an Extended Health and Disease Model

**DOI:** 10.3389/fpsyg.2021.723884

**Published:** 2021-09-13

**Authors:** Ian W. Listopad, Tobias Esch, Maren M. Michaelsen

**Affiliations:** Institute for Integrative Health Care and Health Promotion, Faculty of Health, Department of Medicine, Witten/Herdecke University, Witten, Germany

**Keywords:** burnout, work engagement, perceived meaningfulness of work, sense of homeliness, spirituality, work culture, bio-psycho-socio-spirito-cultural model

## Abstract

Apart from biological, psychological, and social factors, recent studies indicate that spirituality and work culture also play an important role in the onset of burnout. Hence, the commonly applied bio-psycho-social model of health and disease might not be sufficient to comprehensively explain and describe burnout. This study empirically investigates the relationship between spirituality (operationalized by perceived meaningfulness of work) and work culture (operationalized by sense of homeliness of the working environment) with burnout risk and work engagement. For this purpose, an anonymous cross-sectional data collection with fully standardized questionnaires and selected socio-demographic and work-related items was conducted among working adults (*n* = 439) from different industries *via* social media and local health service centers. For all scales and subscales, we found significant moderate to strong correlations. Furthermore, positive meaning within the perceived meaningfulness of work scale was the largest beta coefficient for burnout (β = −0.65) and work engagement (β = 0.62). Within sense of homeliness, the largest beta coefficient for burnout was needs fulfillment (β = −0.34) and work engagement emotional connection (β = 0.36). The strong associations suggest that the current health and disease model needs to be expanded to a bio-psycho-socio-spirito-cultural model to be able to sufficiently describe burnout. The perceived meaningfulness of work and a sense of homeliness should be adequately considered when examining the onset of burnout, describing burnout as a concept, and explaining work engagement.

## Introduction

In the modern world, work is currently characterized by various changes such as increasing complexity, dynamics, digitalization, and changing values (Ameln and Wimmer, 2016). These circumstances have increased stress levels among working individuals and the general population (Hapke et al., 2013; DAK, 2015; De Beer et al., 2016; Siegrist and Li, 2018). Psychological research shows that chronic stress can result in disorders, such as depressive symptoms, anxiety, sleep, and cardiovascular disorders, neurological and immunological diseases, or burnout (Esch et al., 2002a,b,c; Hapke et al., 2013; Kivimäki et al., 2018; Koutsimani et al., 2019). Following the model of allostatic load/allostatic stress response (McEwen, 1998), which combines both biomedical models, i.e., auto regulatory reaction model of the organism and psychological theories (perception, processing, and coping with potentially stressful stimuli) (Esch, 2003; Esch and Stefano, 2010; Werdecker and Esch, 2018), current research focuses on the self-regulation aspect of the organism (maintaining stability through changes), i.e., allostasis. According to the theory of allostatic load, a permanent or long-lasting stimulation (chronic excitation) by a stressor without sufficient recovery phases can lead to an overstrain of the organism. The stress reaction (response) affects all important organ systems, which means that a self-regulation disturbance (or perturbation) has effects on various aspects of wellbeing (Esch and Stefano, 2007, 2010; Esch, 2008a, 2011b; Park et al., 2013, Thoits, 2010; Fragoso et al., 2016; Kronenberg et al., 2017; Werdecker and Esch, 2018). Chronic stress represents a risk that can also be associated with various physical (e.g., Maslach et al., 2001; Esch et al., 2002a,b,c), psychosomatic (Melamed et al., 2006), and mental disorders such as depression and anxiety (Esch et al., 2002c; Hapke et al., 2013; Koutsimani et al., 2019). These symptoms are also attributed to the burnout syndrome (e.g., Melamed et al., 2006; Hapke et al., 2013; Koutsimani et al., 2019). Hence, burnout is associated with various forms of job dissatisfaction, lower productivity, disengagement, an increased intention to quit, and absenteeism (Maslach et al., 2001; Maslach and Leiter, 2008; Alarcon, 2011; Kim and Kao, 2014).

According to Esch (2017, 2019), burnout can be characterized as a state in which an individual no longer experiences oneself as authentic or “resonant,” i.e., what happens outside (e.g., at work, household, and the care of relatives) is no longer in accordance with what is felt inside. As a result, individuals no longer experience themselves as coherent and harmonious because what is important to them has no correspondence in their environment. Consequently, Esch (2019) attributes a central role to the feeling of controllability and perceived meaningfulness of work (experience or *sense* of meaningfulness vs. an externally derived or a received *attribution* of meaningfulness, such as a prestigious job) and argues for extending the established health and disease model by Engel (1977) to be able to holistically describe burnout. He suggests to include a “*spirito-cultural semantic dimension*” (subjective dimension^1^), which consist of a *spiritual* (e.g., perceived meaningfulness, belief, and faith) and *cultural sub-dimension* (e.g., a sense of homeliness, belongingness, and connectedness) within a bio-psycho-socio-spirito-cultural context. In this context, there is evidence that religiosity/spirituality, faith, and belief (Esch, 2008a, 2010, 2011a,b; Doolittle et al., 2013; Kim and Yeom, 2018; Carneiro et al., 2019), meaningfulness (Daniel, 2014; Fragoso et al., 2016; Esch, 2019; Scanlan and Hazelton, 2019), and the sense of coherence, which consists of the components meaningfulness, manageability, and understandability, are associated with low levels of burnout or stress and can promote wellbeing and mental health (Van der Westhuizen et al., 2015). In addition, preliminary research suggests that community (Maslach and Leiter, 2008; Cicognani et al., 2009), value congruence (Lindblom et al., 2006; Asensio-Martínez et al., 2017), and effort–reward imbalance (high ERI ratio) are also negatively associated with burnout and stress (Basińska and Wilczek-Ruzyczka, 2013; Tang et al., 2018). Consequently, according to Esch (2019), from a healthcare perspective, the description of burnout needs to be expanded to avoid evasive or vague designations as it occurs in the International Classification of Diseases (ICD-11, Hillert et al., 2020; WHO, 2021), where burnout is defined as an “occupational phenomenon,” and to properly and holistically describe, diagnose, treat, and prevent burnout (Esch, 2019). Based on various studies (e.g., Cicognani et al., 2009; Daniel, 2014; Scanlan and Hazelton, 2019), there are indications that the factors^2^ that cannot be clearly assigned to the bio-psycho-social model of health and disease are also related to the syndrome. This means that the onset of burnout could also be explained by factors outside the three-dimensional health and disease model.

Thus, the objective of the present study is to advance our understanding of the onset and description of burnout and work engagement by empirically investigating the role of spirituality and work culture based on a model of health and disease. This study addresses a central question on this basis (objective):

*Research question: What is the role of spirituality and work culture in the context of burnout and work engagement?*

Thus, we aim to make four contributions. Firstly, we examine the assumption whether spirituality can be added as a sub-dimension within the health and disease model by testing the relationship between perceived meaningfulness of work and burnout. Secondly, we investigate the association between perceived meaningfulness of work and work engagement as a possible “antipode” of burnout. Thirdly, we investigate whether work culture can be added as an additional sub-dimension within the model. For this purpose, we investigate the relationship between a sense of homeliness of the working environment and burnout. Fourthly and finally, we also examine the relationship between work culture and work engagement.

### Theoretical Background and Hypotheses Development

#### Background of Burnout and Work Engagement

The term “burnout” was used first in the 1970s in the USA when the phenomenon began to be observed with a certain regularity (Maslach et al., 2001). Burnout was observed first among health professionals (Maslach and Jackson, 1981; Alarcon et al., 2009). Further research has shown that burnout also occurs in other occupational groups and industries (e.g., Fusilier and Manning, 2005; Cicognani et al., 2009; Van der Westhuizen et al., 2015; Van den Broeck et al., 2017; Koutsimani et al., 2019; Rajendran et al., 2020). Although Burisch (2014) identified 129 different burnout symptoms, emotional exhaustion (EE) is still considered as a key aspect of burnout (Kristensen et al., 2005; Shirom and Melamed, 2006; Maslach and Leiter, 2008; Seidler et al., 2014). According to WHO (2021), burnout is described by the three subscales^3^ of the Maslach Burnout Inventory (MBI; Maslach et al., 1996) in the ICD-11. The subscale (i) *EE* describes the feeling of overstrain and exhaustion of one's own emotional and physical resources. The (ii) *cynicism* (CY) (*or depersonalization*) subscale refers to the interpersonal-contextual component of burnout, where negative, emotionless, or overly distanced reactions to various aspects of work may occur. The (iii) *reduced professional efficacy (rPE)/personal accomplishment (or ineffectiveness)* subscale refers to the feeling of indecision and self-rated decline in productivity at work (Maslach et al., 2001; Maslach and Leiter, 2008). The significance of the results of these three subscales is obtained from the fact that the syndrome clearly integrates the experience of stress of an individual into the social context of the workplace (Maslach, 1993; Maslach and Leiter, 2008). Although there are other instruments for measuring the burnout syndome, in addition to the MBI [e.g., Copenhagen Burnout Inventory by Kristensen et al. (2005); Oldenburg Burnout Inventory by Demerouti et al. (2001); and Shirom-Melamed Burnout Measure by Shirom and Melamed (2006)], its use in research and practice predominates (Korczak and Huber, 2012).

In addition to Maslach's burnout construct, the concept of work engagement was developed (Schaufeli and Bakker, 2004) and described by some authors as a positive antipode or antithesis of burnout (Schaufeli and Bakker, 2004; Maslach and Leiter, 2008). As work engagement and its subscales [vigor (VI), dedication (DE), and absorption (AB)] complement the burnout construct, it has already been used in various cross-sectional and longitudinal studies on the development of burnout (e.g., Schaufeli and Bakker, 2004; Narainsamy and van der Westhuizen, 2013; Bakker et al., 2014; Chan et al., 2015; Möltner et al., 2017; Van den Broeck et al., 2017). The simultaneous investigation of burnout and work engagement allows for differentiated analyses as they have different consequences for employees and organizations and may be associated with different intervention strategies (Schaufeli and Bakker, 2004). There are preliminary indications that burnout is associated with high job demands and (to a lower extent) low job resources, whereas work engagement, on the other hand, is related to job resources (Bakker et al., 2007, 2014; Van den Broeck et al., 2017). Although both, burnout and work engagement, are related to work-related outcomes, burnout is more strongly associated with health consequences for employees, whereas work engagement is more strongly associated with motivation. Consequently, both concepts provide similar but distinct insights at individual and organizational levels (Schaufeli and Bakker, 2004; Bakker et al., 2014).

#### Limitations of the Bio-Psycho-Social Model

Currently, the bio-psycho-social model by Engel (1977) is typically used to explain the development of health and disease as complex interactions within the person (biological/physical and psychological factors) and the environment (workplace-related, social, or economic factors) involved in the development of health and illness (Borrell-Carri et al., 2004; Han, 2008; Berger et al., 2012). According to various studies, the current model of health and disease has limitations. For example, there is an isolation (demarcation) between the biological, psychological, and social dimensions (Suls and Rothman, 2004; Babalola et al., 2017; Lehman et al., 2017), a lack of consideration for the emotional relationships between patients and professionals (nurses, doctors, etc.) (Havelka et al., 2009), dominance of the biological dimension within practice, and a lack of cultural and spiritual aspects (Esch, 2008a, 2011a, 2019).

#### Burnout Factors

The onset of burnout has commonly been described within the bio-psycho-social model of health and disease (e.g., Weber and Jaekel-Reinhard, 2000; Han, 2008; Seemüller et al., 2019). In this context, factors such as high workload (Maslach and Leiter, 2008; Asensio-Martínez et al., 2017), low control at work (Brouwers et al., 2011), and high work demands [according to the job strain model/demand control model by Karasek (1979), Demerouti et al. (2001) and Kivimäki et al. (2006)] are mentioned in various studies. Furthermore, role ambiguity (Maslach and Leiter, 2008; Vassos and Nankervis, 2012), private support or collegial support (Baruch-Feldman et al., 2002, Charoensukmongkol et al., 2016), work–family conflict (Blundson et al., 2006; Brouwers et al., 2011; Vassos and Nankervis, 2012), coping strategies (Shin et al., 2014; Mattei et al., 2017), low recovery/low psychological detachment (Sonnentag et al., 2010; Sonnentag, 2018), physical activities (Dreher et al., 2018), and effort–reward imbalance (Schulz et al., 2009; Basińska and Wilczek-Ruzyczka, 2013; Jachens et al., 2019) have been investigated in association with burnout. Additional factors such as the Big Five personality traits (Castillo-Gualda et al., 2019), resilience (Nevill and Havercamp, 2019), self-esteem, self-efficacy (Alarcon et al., 2009), and perfectionistic concerns (Rice and Liu, 2020) are also associated with burnout.

In addition to these factors, there is initial evidence that the bio-psycho-social model of Engel (1977) is not sufficient to assign all factors related to burnout to the current version of the health and disease model and thus to describe the pathogenesis of the syndrome in a holistic manner. Various identified factors (e.g., perceived meaningfulness and a sense of homeliness) that have previously received little or no attention in burnout research also seem to be associated with (chronic) stress or burnout (Van Dierendonck et al., 2005; Cicognani et al., 2009; Doolittle et al., 2013; Ivtzan et al., 2013; Daniel, 2014; Fragoso et al., 2016; Esch, 2017, 2019; Van Wingerden and van der Stoep, 2017). Thus, investigations have also suggested that the phenomenon of burnout may gain a broader understanding in the context of spiritual factors (Van Dierendonck et al., 2005; Doolittle et al., 2013; Ivtzan et al., 2013; Daniel, 2014; Fragoso et al., 2016; Esch, 2017, 2019; Van Wingerden and van der Stoep, 2017) and work culture, or the sense of “feeling at home” at the workplace (e.g., through sharing the same values) (Cicognani et al., 2009; Asensio-Martínez et al., 2017).

#### Hypotheses Development

In the context of explaining the development of burnout and the factors related to work engagement, the aspects such as spirituality and work culture currently present a research gap.

##### Spirituality in the Context of Burnout and Work Engagement

There are various phenomena that can be assigned to a broader concept of spirituality, such as perceived meaningfulness, faith, belief, meditation, and mindfulness (Esch et al., 2006; Esch and Stefano, 2007, 2010; Esch, 2008a, 2010, 2011a, 2017, 2019). A negative relationship between these factors and burnout or stress has already been demonstrated in initial studies (e.g., Levert et al., 2000; Ernst et al., 2009; Kim and Yeom, 2018; Carneiro et al., 2019). Kinnunen et al. (2020) demonstrated that mindfulness-based interventions are associated with reduced burnout in long term. Furthermore, it was observed that employees who are more spiritually or religiously oriented have greater resilience and therefore suffer less from burnout (Kim and Yeom, 2018; Carneiro et al., 2019). There is also preliminary evidence for a positive relationship between a low sense of coherence [consisting of the sense of comprehensibility, manageability, and meaningfulness (Antonovsky, 1996) and a higher risk of burnout (Levert et al., 2000)]. In this study, perceived meaningfulness is considered as a central aspect of spirituality. Negative correlations between perceived meaningfulness of work and burnout (Daniel, 2014; Fragoso et al., 2016; Van Wingerden and van der Stoep, 2017), as well as between perceived meaningfulness of work and wellbeing (Soane et al., 2013), have already been reported in several studies. Additional evidence was provided by several empirical investigations, identifying positive correlations between perceived meaningfulness of work and work engagement (Soane et al., 2013; Fragoso et al., 2016; Van Wingerden and van der Stoep, 2017). In Van Wingerden and van der Stoep (2017), a quantitative survey of employees revealed strong correlations between perceived meaningfulness and burnout, as well as between perceived meaningfulness and work engagement among participants from different organizations and professions. Given the initial associations and comparatively limited research in this area, we decided to examine perceived meaningfulness of work as a burnout factor that can be assigned to the dimension of spirituality (in an expanded health and disease model).

Moreover, a differentiated consideration of the subscales of burnout, perceived meaningfulness of work, the application to the current burnout concept by Maslach et al. (1996) and the integration of the results within a health and disease model still reveal research gaps. Thus, based on these theoretical constrcuts and empirical findings and the existing research claims, the following hypotheses have been formulated:

*Hypothesis 1a: A greater sense of perceived meaningfulness of work is negatively associated with burnout risk*.

*Hypothesis 1b: A greater sense of perceived meaningfulness of work is positively associated with work engagement*.

##### Work Culture in the Context of Burnout and Work Engagement

Changes in the world of work and new emerging job forms (World Economic Forum, 2017) are not only accompanied by a flexibilization of work in terms of time and place but also by an increased delimitation of work (Ameln and Wimmer, 2016). At the same time, the sense of being at home within the working environment is associated with increased individual wellbeing and mental health (Farrell et al., 2004; Pretty et al., 2006; Plys and Qualls, 2019). According to Esch (2017, p. 148), the feeling of “inner home” and “connectedness” is possibly biologically and culturally anchored in the limbic system of the brain. According to McMillan and Chavis (1986), the perceived sense of being “at home” (or the sense of community) within an environment consists of a sense of group membership, influence, integration and needs fulfillment (NF), and emotional connectedness. A 2009 study (Cicognani et al., 2009) identified a negative relationship between the sense of community and burnout. Based on the initial evidence, the immediacy of perceived homeliness, and comparably limited research in this area, we decided to examine perceived homeliness at work as a further burnout factor that can be assigned to the work culture dimension (within an extended health and disease model).

In this context, there are still research gaps concerning the relationship between a sense of homeliness in a working environment and burnout as well as the consideration of corresponding subscales, the transfer to the current burnout concept of Maslach et al. (1996), and the integration of the concept into a comprehensive health and disease model. Based on this theoretical and empirical evidence, the following hypotheses have been formulated:

*Hypothesis 2a: A greater sense of homeliness within the working environment is negatively associated with the risk of burnout*.

*Hypothesis 2b: A greater perceived sense of homeliness within the working environment is positively associated with work engagement*.

Consequently, this study investigates relationships between perceived meaningfulness, sense of homeliness and burnout, as well as between perceived meaningfulness, sense of homeliness and work engagement, and thus demonstrates which burnout factors exist outside the current health and disease model.

## Materials and Methods

### Research Design

We chose a cross-sectional design because this research design has been used in other studies with similar questions (e.g., Cicognani et al., 2009; Van Wingerden and van der Stoep, 2017; Van Wingerden et al., 2018b), and we considered our study as initial in the context of the extended health and disease model. Although we are not aware of any study to date that has deliberately chosen to sample healthy and mentally ill working individuals, we chose to collect data in this way to increase the variance in burnout levels. Thus, the sampling approach was based on the results of several studies that have reported that burnout is associated with various disorders such as depression, anxiety, or various personality disorders (e.g., Schwarzkopf et al., 2016; Bianchi et al., 2018; Koutsimani et al., 2019), so we assumed higher levels of burnout in healthcare centers than outside these centers.

### Sample Size and Power Calculations

We computed power calculations to determine the necessary minimum sample size using the statistical program G-Power (Faul et al., 2007). We calculated that at least 340 participants were required to determine an effect size [based on previous studies with comparable theoretical constructs and hypotheses (McCarthy et al., 1990)] of 0.158 (Cohen's *d*) with a power of 0.8 and a significance level of 95% (α = 0.05).

### Procedures and Participants

Participation in the survey was completely voluntary and anonymous. Respondents received no financial or material compensation for their contribution. The survey took approximately 8–12 min to complete.

The study pursued two recruitment strategies. Participants were recruited online *via* social media channels (Facebook, LinkedIn, XING, and Twitter) as well as through direct contact (*via* e-mail) with companies from the education, healthcare, consulting, and chemical industries and were invited to take part in an online survey (*n* = 255). In addition, a paper-and-pencil survey was conducted in seven psychotherapeutic (joint) practices (*n* = 128), four medical care centers (*n* = 31), and one hospital for interdisciplinary psychosomatics and psychiatry (*n* = 25). All these centers are based in Germany. The latter were chosen to generate a broader spectrum of burnout-levels as we expected generally lower levels among online recruited participants and higher levels among those recruited in health centers. This means that the sample also includes participants with mental illnesses (e.g., depression and anxiety). Here, patients were invited by staff to participate in the survey. In addition, potential participants in practices and medical care centers were recruited *via* table displays with a short introduction and invitation to participate in the study. Anonymity was ensured by providing opaque envelopes with each questionnaire. The convenient sampling was also chosen because we did not want to measure or identify burnout but wanted to examine relationships with the corresponding (sub-)dimensions in the burnout sample. The data collection took place between September 2019 and February 2020. A total sample size of 439 participants was recruited^4^. A prerequisite for participation in the survey was a minimum age of 18 years and a working week of at least 6 h (regardless of holidays or sick leave). In accordance with the ICD-11, which describes burnout as an “occupational phenomenon” (WHO, 2021), we considered only working individuals.

### Sample Description

To gain a detailed picture of our sample, 13 socio-demographic and work-related items [gender, age, work experience, marital status, the highest level of education, vocational education (multiple selection possible), sector affiliation (multiple selection possible), type of employment, professional responsibility, company size, tenure (years with the company), average weekly working hours, and contractually agreed weekly working hours] were collected. Age, professional experience, company size, tenure, current average weekly working hours, and contractually agreed weekly working hours were measured using predefined ranges. The characteristics of the study population are presented in Table 1. Details of the aggregated study population considered in the data analysis are presented below.

**Table 1:** Baseline characteristics of the study population (*n* = 439).

**Characteristics**	***n* (%)**	**Characteristics**	***n* (%)**
**Age**		**Work experience**	
18–24 years	35 (8%)	0–5 years	131 (29,8%)
25–35 years	180 (41%)	6–10 years	77 (17,5%)
36–46 years	102 (23%)	11–15 years	65 (14,8%)
47–57 years	92 (21%)	16–20 years	37 (8,4%)
≥58 years	30 (6,8%)	21–25 years	29 (6,6%)
		26–30 years	44 (10%)
**Sex**		≥31 years	56 (12,8%)
Female	280 (63.8%)		
Male	158 (36%)	**Average weekly working hours**	
Other	1 (0.2%)	6–10 h	16 (3.6%)
		11–20 h	39 (8.9%)
**Marital status**		21–30 h	74 (16.9%)
Single	120 (27.3%)	31–40 h	165 (37.6%)
In partnership, living together	99 (22.6%)	41–50 h	101 (23%)
In partnership, living separated	37 (8.4%)	51–60 h	28 (6.4%)
Married, living together	151 (34.4%)	≥61 h	16 (3.6%)
Married, separated living	9 (2.1%)		
Other (e.g., widowed, divorced)	23 (5.2%)	**Contractually agreed weekly working h**	
		6–10 h	10 (2,3%)
**Company size (number of employees)**		11–20 h	56 (12,8%)
≤ 10	59 (13.4%)	21–30 h	76 (17,3 %)
11–20	52 (11.8%)	31–40 h	262 (59,7%)
21–199	97 (22.1%)	≥41 h	35 (8%)
200–999	73 (16.6%)		
1,000–10,000	81 (18.5%)	**Professional responsibility**	
10,000–50,000	34 (7.7%)	Without leadership responsibility	329 (74,9%)
≥50.000	43 (9.8%)	With leadership responsibility	110 (25,1 %)
**Highest level of education**		**Sector affiliation (multiple selection)**	
No degree	3 (0.7%)	Automobile	11 (2,5%)
Secondary modern school (year 5 to 9 in Germany)	23 (5.2%)	Banking and finance	17 (3,9%)
Junior high school (year 5 to 10 in Germany)	86 (19.6%)	Education	48 (10,9%)
General qualification for university entrance	113 (25.7%)	Chemistry and pharma	18 (4,1%)
Academic degree	200 (45.6%)	Consulting and services	88 (20%)
Other (e.g., doctorate / Ph.D., habilitation)	14 (3,2%)	Electronic data processing and IT	40 (9,1%)
		Energy and environment	6 (1,4%)
**Type of employment**		Health and social affairs	127 (28,9%)
Clerk	309 (70,4%)	Trade and commerce	32 (7,3%)
Worker	37 (8,4%)	Handicraft	20 (4,6%)
Apprentice	14 (3,2%)	Manufacturing	30 (6,8%)
Public official	37 (8,4%)	Culture and Events	12 (2,7%)
Intern	2 (0,5%)	Public service	52 (11,8%)
Self-employed	40 (9,1%)	Textile and Fashion	5 (1,1%)
		Traffic, Transport and Logistics	17 (3,9%)
**Company size (number of employees)**		Science	17 (3,9%)
≤ 10	59 (13,4%)	Another sector	34 (7,7%)
11–20	52 (11,8%)		
21–199	97 (22,1%)	**Tenure (years with the company)**	
200–999	73 (16,6%)	≤ 5 years	242 (55,1%)
1.000–10.000	81 (18,5%)	6–10 years	80 (18,2%)
10.000–50.000	34 (7,7%)	11–15 years	43 (9,8%)
≥50.000	43 (9,8)	16–20 years	31 (7,1%)
		21–25 years	15 (3,4%)
**Vocational education (multiple selection possible)**		26–30 years	14 (3,2 %)
No vocational education	111 (25.3%)	≥31 years	14 (3,2 %)
Vocational education (in-plant training)	152 (34.6%)		
Vocational qualification (vocational school education)	121 (27.6%)		
Completion of a professional school, technical school, master school,administration and business academy or specialized academy	93 (21.2%)		

Of the 439 participants who took part in the survey, 280 (63.8%) were women. Consequently, similar to other studies in the field, there is a surplus of women in our sample (e.g., Van Wingerden and van der Stoep, 2017; Van Wingerden et al., 2018b; Scanlan and Hazelton, 2019).

The predefined age ranges were 18–24 years (*n* = 35; 8%), 25–35 years (*n* = 180; 41%), 36–46 years (*n* = 102; 23%), 47–57 years (*n* = 92; 21%), and 58 years or older (*n* = 30; 6.8%). Compared to previous studies among employees and the total working population, we identified no abnormalities regarding the distribution of age (e.g., Maslach and Leiter, 2008; Van Wingerden and van der Stoep, 2017; Scanlan and Hazelton, 2019).

Furthermore, 120 (27.3%) of the participants were single, 99 (22.6%) were living together in a non-marital partnership, 37 (8.4%) were in a partnership living separated, 151 (34.4%) were married and living together, 9 (2.1%) were married but living separately, and 23 (5.2%) were included in the group “others” (e.g., divorced and widowed). Compared to the total working population, the sample appears to be representative (e.g., ESE, 2017).

Of all participants, 3 (0.7%) had no school degree, 23 (5.2%) had a secondary modern school degree (from year 5–9 in Germany), 86 (19.6%) had a secondary modern school/junior high school degree (from year 5–10 in Germany), 113 (25.7%) had a general qualification for university entrance (*Abitur*), 200 (45.6%) participants had an academic degree as the highest education, whereas 14 (3.2%) had a doctorate/Ph. D. or a habilitation. In this context, it is noticeable that many of our respondents have an academic degree although this unequal distribution can also be seen in other similar studies (e.g., Van Wingerden and van der Stoep, 2017; Van Wingerden et al., 2018a,b).

In addition, we asked the participants about the size of the company (number of employees) in which they were primarily employed. The ranges of the company size were 1–10 employees (*n* = 59; 13.4%), 11–20 employees (*n* = 52; 11.8%), 21–199 employees (*n* = 97; 22.1%), 200–999 employees (*n* = 73; 16.6%), 1,000–10,000 employees (*n* = 81; 18.5%), 10,000–50,000 employees (*n* = 34; 7.7%), and more than 50,000 employees (*n* = 43; 9.8%). Although there are no major differences in the distribution of employees in different companies, in our sample more people are employed in larger company sizes compared to the overall European population (ESE, 2020).

Average weekly working time ranges were 6–10 h (*n* = 16; 3.6%), 11–20 h (*n* = 39; 8.9%), 21–30 h (*n* = 74; 16.9%), 31–40 h (*n* = 165; 37.6%), 41–50 h (*n* = 101; 23%), 51–60 h (*n* = 28; 6.4%), and 61 h and more (*n* = 16; 3.6%). In addition to the average weekly working hours, we asked the participants about their contractually agreed upon working hours. The time intervals for this were 10 h or less (*n* = 10; 2.3%), 11–20 h (*n* = 56; 12.8%), 21–30 h (*n* = 76; 17.3%), 31–40 h (*n* = 262; 59.7%), and 41 h and more (*n* = 35; 8%). A striking observation is that 8% of the participants had a contractually agreed weekly working time of 40 h or more, but in total 33% of the respondents worked 40 h or more. Hence, there is a trend toward overtime.

Considering that our study is the first to examine burnout syndrome based on an extended health and disease model, we decided to neglect the focus on a specific industry and thus cover different settings. Because participants may also have more than one job, we allowed multiple responses. Thus, the participants in our sample are distributed across the following industries: automobile (*n* = 11; 2.5%), banking and finance (*n* = 17; 3.9%), education (*n* = 48; 10.9%), chemistry and pharma (*n* = 18; 4.1%), consulting and services (*n* = 88; 20%), electronic data processing and IT (*n* = 40; 9.1%), health and social affairs (*n* = 127; 28.9%), trade and commerce (*n* = 32; 7.3%), handicraft (*n* = 20; 4.6%), manufacturing (*n* = 30; 6.8%), culture and events (*n* = 12; 2.7%), textile and fashion (*n* = 5; 1.1%), traffic, transport and logistics (*n* = 17; 3.9%), science (*n* = 17; 3.9%), and another sector (*n* = 34; 7.7%). In addition, 329 participants (74.9%) without management leadership responsibility and 110 participants (25.1%) with leadership responsibility took part in our study.

### Measures

Cross-sectional data were collected using fully standardized questionnaires, which were comprised of 5 sections with a total of 55 items and were provided in German and English. In addition to the aforementioned socio-demographic and work-related data, surveys on burnout, work engagement, perceived meaningfulness of work, and sense of homeliness were included. The reliability analysis was based on the calculation of internal consistency (Cronbach's alpha).

#### Burnout

The MBI-General Survey (MBI-GS) in English by Maslach et al. (1996) and in German by Schaufeli et al. (1996) was used to assess burnout. The questionnaire comprises 16 items with a 7-level Likert scale (ranging from 1 = *never* to seven = *every day*). It includes the subscales (i) EE (five items: e.g., “*I feel emotionally drained from my work*”), (ii) CY (five items: e.g., “*I have become less enthusiastic about my work*”), and (iii) rPE (six items: e.g., “*In my opinion, I am good at my job*”—inversely coded). High scores on EE, CY, and rPE are the indicative of a high burnout risk. The reliability analysis showed an acceptable internal consistency of all three subscales of the MBI-GS (EE: α = 0.91; CY: α = 0.90; and rPE: α = 0.83) and for total burnout (α = 0.92).

#### Work Engagement

The Utrecht Work Engagement Scale-9 (UWES-9) by Schaufeli and Bakker (2003) and the German version by Sautier et al. (2015) was used to measure work engagement. The short version of the UWES is a valid and reliable self-assessment instrument for measuring work engagement, which has been used in various research projects (e.g., Sautier et al., 2015; Möltner et al., 2017). The short version comprises nine items with a seven-level Likert scale (ranging from 1 = *never* to seven = *always/every day*). As already mentioned, the questionnaire comprises the subscales (i) VI (three items: e.g., “*At my work, I feel bursting with energy*”), (ii) DE (three items: e.g., “*My job inspires me*”), and (iii) AB (three items: e.g., “*I feel happy when I am working intensely*”). There are no reverse-coded items in this scale. High scores on VI, DE, and AB indicate a high level of work engagement. According to Sonnentag (2003), it was not possible to demonstrate three dimensionality (i.e., VI, DE, and AB as subscales) for the German version, and a distinction between the subscales is not recommended (Schaufeli et al., 2002). Consequently, in this study, we do not differentiate between the three subscales of work engagement in the analyses. We identified the acceptable values indicating reliability for the total work engagement scale (α = 0.94) and for the underlying subscales (VI: α = 0.88; DE: α = 89; and AB: α = 84).

#### Perceived Meaningfulness of Work

This concept was measured using the Work and Meaningful Inventory (WAMI) published in the English version by Steger et al. (2012) and the German version by Harzer (2016). The scale comprises 10 items with five-level Likert scales (ranging from 1 = *absolutely untrue* to 5 = *absolutely true*). The questionnaire comprises the subscale (i) positive meaning (PM) (four items: e.g., “*I have found a meaningful career*”), (ii) meaning making through work (MM) (three items: e.g., “*I view my work as contributing to my personal growth*”), and (iii) greater good motivations (GG) (three items: e.g., “*My work really makes no difference to the world*”; only this item is reverse coded). Perceived meaningfulness of work refers to the individually perceived (i.e., subjective feeling/experience or sense of) meaningfulness within an organization. High scores on PM, MM, and GG indicate a high level of perceived meaningfulness of work (Steger et al., 2012). The level of internal consistency for the total scale perceived meaningfulness of work (α = 0.94) and for the three subscales (PM: α = 0.90; MM: α = 81; and GG: α = 0.80) was considered to be acceptable.

#### Sense of Homeliness

This concept was assessed by using the Brief Sense of Community Scale (BSCS). The English version has been developed by Peterson et al. (2008) and the German version has been developed by Wombacher et al. (2010). This short scale is based on the theoretical considerations of a sense of community of McMillan and Chavis (1986), which we consider in concordance with our definition of homeliness, and it comprises eight items with a five-level Likert scale (ranging from 1 = *strongly agree* to 5 = *strongly disagree*). The questionnaire was originally developed to measure the sense of community in neighborhoods and has not yet been used in an organizational context. The German-language version was also used to measure the sense of community within the German armed forces. We adapted the versions to the workplace by replacing the word “group” with “organization” and “soldiers” with “employees” in the German translation of the questionnaire (Wombacher et al., 2010) and the word “neighborhood” with “organization” in the English version. The subscales (i) NF (two items: e.g., “*I can get what I need in this organization*”), (ii) group membership (GM) (two items: e.g., “*I feel like a member of this organization*”), (iii) Influence (IN) (two items: e.g., “*I have a say about what goes on in my organization*”), and (iv) emotional connection (EC) (two items: e.g., “*I have a good bond with others in this organization*”) are covered. A high level of homeliness is indicated by a high score of NF, GM, IN, and EC. The internal consistency showed acceptable reliability in total (α = 0.91) and in relation to the four subscales (NF: α = 0.90; GM: α = 87; IN: α = 63; and EC: α = 0.64).

### Data Analyses

Statistical analyses were performed using the SPSS Statistics, regression analysis and linear models (RLM) macro (Darlington and Hayes, 2016). The item analysis included the calculation of frequencies, means (*M*), medians (*Mdn*), and *SDs*. To get an overview in terms of strength and direction between two variables, we decided to use bivariate descriptive statistics. Bivariate descriptive statistics include Pearson correlations for the metric variables and Spearman rank correlations for ordinal variables (socio-demographic/work-related variables). A point-biserial correlation was used to measure the strength and direction of the dichotomous variable gender (female = 0; male = 1)^5^. The statistical significance was set to α < 0.05. No correction for multiple testing was applied.

The hypotheses were tested using ordinary least squares (OLS) multiple linear regression as we wanted to consider multiple factors of interest simultaneously in the prediction. We conducted a series of multiple regression analyses to examine the predictors of burnout and work engagement. Multiple regression analyses were conducted separately for burnout and each of the three subscales, as well as for work engagement as the dependent variables, and the included independent variables, which were significant in the correlation analysis. Hypothesized models were tested with and without a set of covariates. The selection of covariates, i.e., (i) age, (ii) gender, (iii) highest level of education, (iv) company size, (v) average weekly working hours, and (vi) marital status, was based on the variables discussed in a few earlier studies regarding their relationship to burnout (Maslach et al., 2001; Bilge, 2006; Soares et al., 2007; Matin et al., 2012; Vassos and Nankervis, 2012; Cañadas-De la Fuente et al., 2015, 2018; Asensio-Martínez et al., 2017; Ezenwaji et al., 2018; Hakanen et al., 2019).

The regression models and dependent variables were tested for violations of normality and regression models were assessed for linearity and homoscedasticity by histogram analysis, probability-probability plots (P-P plots) of regression standardized residual, and residual scatter plots. Regression models exhibiting considerable heteroscedasticity were calculated with HC2-corrected SEs (heteroscedasticity-consistent; Long and Ervin, 2000). As average weekly working hours fulfilled the linearity assumption in each of our regression models, it was incorporated as a continuous covariate.

The overall fit of the regression model was assessed using adjusted *R*-squared (*R*^2^_adj_) and the overall *F*-test. For the individual predictors *B* (regression coefficient), β (standardized coefficient), and *p* (significance) are provided (see Tables A.1–A.10 in the Supplementary Material).

For testing Hypothesis 1a, the contribution to burnout total score, as well as EE, CY, and rPE, was assessed using the independent subscales for perceived meaningfulness of work, i.e., PM, MM, and GG (step 1). Afterwards, the control variables were entered in each regression (step 2). Similarly, to test Hypothesis 1b, the contribution to work engagement was assessed, including the independent subscales for perceived meaningfulness of work (step 1) and, subsequently, the control variables were entered (step 2). To test Hypothesis 2a, we assessed the contribution to the overall burnout score, as well as EE, CY, and rPE, using the independent subscales for a sense of homeliness, i.e., NF, GM, IN, and EC (step 1). Next, the control variables were added to the regressions (step 2). For testing Hypothesis 2b, we assessed the contribution to work engagement using the independent subscales for sense of homeliness, i.e., NF, GM, IN, and EC (step 1). Secondly, we included the control variables (step 2). Because no three-dimensionality could be confirmed for work engagement, and a distinction between the three subscales is not recommended (Schaufeli et al., 2002; Sonnentag, 2003; Sautier et al., 2015), we have ignored subsequent regressions for the work engagement subscales.

## Results

The total burnout scale and the three underlying subscales, as well as the total work engagement were used as dependent variables. Hence, four sets of models were estimated.

### Descriptive Statistics and Correlations

Table 2 shows the descriptive statistics (*M, Mdn*, and *SD*) for the main variables of interest. The univariate descriptive statistics display burnout (*M* = 3.32; *Mdn* = 3.17; and *SD* = 1.23), work engagement (*M* = 4.58; *Mdn* = 4.67; and *SD* = 1.25), perceived meaningfulness of work (*M* = 3.34; *Mdn* = 3.47; and *SD* = 0.98), a sense of homeliness (*M* = 3.54; *Mdn* = 3.75; and *SD* = 0.97), and all corresponding subscales to ensure sample transparency.

**Table 2:** Univariate descriptive statistics.

**Variable**	***M***	***Mdn***	***SD***
Burnout	3.32	3.17	1.23
Emotional exhaustion	4.20	4.20	1.55
Cynicism	3.34	3.00	1.81
Reduced professional efficacy	2.42	2.17	1.05
Work engagement	4.58	4.67	1.25
Vigor	4.57	4.67	1.31
Dedication	4.72	5.00	1.36
Absorption	4.44	4.67	1.36
Perceived meaningfulness of work	3.34	3.47	0.98
Positive meaning	3.56	3.75	1.06
Meaning making through work	3.29	3.33	1.06
Greater good motivations	3.17	3.33	1.11
Sense of homeliness	3.54	3.75	0.97
Needs fulfillment	3.36	3.50	1.17
Group membership	3.74	4.00	1.19
Influence	3.31	3.50	1.10
Emotional connection	3.74	4.00	1.00

*n = 439; M (mean); Mdn (median); SD*.

Table 3 shows the correlations for burnout, work engagement, perceived meaningfulness of work, and sense of homeliness, as well as all subscales. We have observed significant correlations between perceived meaningfulness of work and burnout (*r* = −0.64), perceived meaningfulness of work and work engagement (*r* = 0.65), and between all subscales. Likewise, we have observed significant correlations between sense of homeliness and burnout (*r* = −0.70), between sense of homeliness and work engagement (*r* = 0.68), and correspondingly also between all subscales.

**Table 3:** Pearson correlations between the study variables.

**Variable**	***r***															
	2	3	4	5	6	7	8	9	10	11	12	13	14	15	16	17
1. Burnout	0.84	0.91	0.72	−0.78	−0.74	−0.75	−0.68	−0.64	−0.69	−0.61	−0.47	−0.70	−0.66	−0.64	−0.48	−0.63
2. Emotional exhaustion	–	0.63	0.40	−0.60	−0.60	−0.53	−0.52	−0.42	−0.46	−0.42	−0.27	−0.52	−0.54	−0.47	−0.32	−0.47
3. Cynicism		–	0.56	−0.72	−0.67	−0.72	−0.62	−0.65	−0.71	−0.59	−0.49	−0.67	−0.63	−0.63	−0.45	−0.61
4. Red. professional efficacy			–	−0.62	−0.57	−0.62	−0.54	−0.53	−0.53	−0.50	−0.41	−0.54	−0.46	−0.50	−0.44	−0.47
5. Work engagement				–	0.93	0.93	0.93	0.65	0.70	0.64	0.46	0.68	0.60	0.62	0.48	0.66
6. Vigor					–	0.79	0.79	0.55	0.60	0.55	0.37	0.63	0.56	0.60	0.42	0.62
7. Dedication						–	0.79	0.71	0.74	0.67	0.54	0.66	0.60	0.59	0.51	0.61
8. Absorption							–	0.55	0.59	0.55	0.37	0.59	0.51	0.54	0.42	0.60
9. Perceived meaningfulness of work								–	0.94	0.92	0.89	0.59	0.55	0.50	0.49	0.52
10. Positive meaning									–	0.84	0.74	0.62	0.57	0.54	0.47	0.55
11. Meaning making through work										–	0.68	0.58	0.54	0.49	0.46	0.53
12. Greater good motivations											–	0.43	0.39	0.35	0.40	0.35
13. Sense of homeliness												–	0.89	0.90	0.80	0.88
14. Needs fulfillment													–	0.75	0.60	0.72
15. Group membership														–	0.61	0.76
16. Influence															–	0.58
17 Emotional connection																–

*n = 439; All correlations were statistically significant at p < 0.01 level (two-tailed); r = r-value*.

Table 4 presents the correlations for selected socio-demographic variables that have been discussed in the literature or which were partially inconsistent in terms of their relationships to burnout. We found significant correlations for age (rPE, perceived meaningfulness of work, PM, and MM). For gender, there was one significant correlation (in our sample, men tend to score slightly higher on NF). Moreover, we identified significant correlations for the highest level of education (CY, work engagement, VI, DE, AB, perceived meaningfulness of work, PM, MM, and GM), and for work experience (rPE and PM). We found the most significant correlations for company size (burnout, CY, work engagement, DE, perceived meaningfulness of work, PM, MM, GG, sense of homeliness, GM, IN, and EC). Finally, we identified significant correlations for weekly working hours (work engagement, VI, AB, and EC).

**Table 4:** Correlations of selected socio-demographic and work-related variables with study variables.

**Variables**	**Age**	**Gender^a^**	**Highest level of education**	**Company size**	**Weekly working hours**
Burnout	−0.01	−0.04	−0.09	0.11^*^	−0.01
Emotional exhaustion	0.03	−0.06	−0.06	0.04	−0.00
Cynicism	0.00	−0.00	−0.10^*^	0.14^**^	0.00
Reduced professional efficacy	−0.10^*^	−0.07	−0.02	0.09	−0.05
Work engagement	0.02	0.04	0.12^*^	−0.11^*^	0.14^**^
Vigor	0.02	0.03	0.12^*^	−0.09	0.13^**^
Dedication	0.02	0.07	0.11^*^	−0.15^**^	0.08
Absorption	0.03	0.00	0.10^*^	−0.07	0.16^**^
Perceived meaningfulness of work	0.11^*^	0.04	0.12^*^	−0.22^**^	−0.02
Positive meaning	0.10^*^	−0.05	0.11^*^	−0.24^**^	−0.00
Meaning making through work	0.12^*^	−0.04	0.11^*^	−0.21^**^	−0.02
Greater good motivations	0.09	−0.03	0.08	−0.17^**^	−0.03
Sense of homeliness	0.03	0.04	0.05	−0.21^**^	0.05
Needs fulfillment	−0.00	0.10^*^	0.05	−0.09	0.01
Group membership	0.01	0.00	0.11^*^	−0.13^**^	0.09
Influence	0.04	0.03	−0.06	−0.35^**^	−0.03
Emotional connection	0.04	0.01	0.05	−0.16^**^	0.10^*^

*n = 439;*

* *p < 0.05;*

** *p < 0.01 (two-tailed);*

a *Gender: 0 = female, 1 = male; one participant (“other”) was not included in the calculation*.

*Spearman rank correlations for ordinal variables*.

### Multiple Regression Analysis

Multiple regression analysis was used to investigate the relationships between the independent variables (i.e., perceived meaningfulness of work and sense of homeliness) and various socio-demographic/work-related variables (as control variables) to the criteria variables (i.e., burnout and work engagement). To make differentiated statements on burnout, additional analyses with the corresponding burnout subscales as dependent variables were performed.

### Hypotheses Testing

In the first set of regressions, we tested the hypotheses that a greater sense of perceived meaningfulness of work is negatively associated with the risk of burnout (Hypothesis 1a) and positively associated with work engagement (Hypothesis 1b). To test both hypotheses similarly, we used the multiple regression analysis with perceived meaningfulness of work as an independent variable and burnout with the corresponding subscales and work engagement as criteria variables. Then, in the second set of regressions, we examined the associations after adding the six control variables (age, gender, highest level of education, company size, average weekly working hours, and marital status).

### Hypotheses Testing for Perceived Meaningfulness of Work and Burnout, and Perceived Meaningfulness of Work and Work Engagement

#### Burnout

Table A.1 (see the Appendix in the Supplementary Material) shows the results of both regression analyses (with and without controls). First, we found significant associations between the dependent variable burnout and the independent variable PM (*B* = −0.76 [*SE* = 0.09]; β = −0.65; *p* < 0.01), MM (*B* = −0.16 [*SE* = 0.08]; β = −0.14; *p* = 0.05]), and GG (*B* = 0.12 [*SE* = 0.06]; β = 0.10; *p* < 0.05). This means that each increase by one unit in the independent variable PM corresponds to a decrease of 0.76 in the risk of burnout. Likewise, an increase of MM by one unit corresponds to a decrease of risk of burnout by 0.16. An increase of GG by one unit corresponds to an increase of burnout risk by 0.12. The principle of interpretation can be applied analogously to the other results. In the second regression, there were no significant changes by adding the covariates regarding the associations between the independent variables PM and GG on the dependent variable total burnout. An exception to the first regression, however, was that the association between the independent variable MM and the dependent variable burnout was not significant (*B* = −0.16 [*SE* = 0.09]; β = −0.14; *p* > 0.05).

#### Emotional Exhaustion

The results of these two regressions are shown in Table A.2. Firstly, our analysis demonstrated significant associations for the independent variables PM (*B* = −0.64 [*SE* = 0.14]; β = −0.44; *p* < 0.01) and GG (*B* = 0.22 [*SE* = 0.09]; β = 0.16; *p* < 0.05) with the dependent variable EE. A significant association between the independent variable MM and the dependent variable EE could not be identified (*B* = −0.24 [*SE* = 0.12]; β = −0.16; *p* > 0.05). Secondly, we found no differences regarding the significant associations between PM, GG, and EE. One noticeable change, however, was the significant association between MM and EE (*B* = −0.26 [*SE* = 0.12]; β = −0.18; *p* < 0.01).

#### Cynicism

Table A.3 presents the results of two regressions. In the first model, we identified a significant relationship between the dependent variable CY and the independent variable PM (*B* = −1.27 [*SE* = 0.12]; β = −0.74; *p* < 0.01), whereas the associations between independent variables MM (*B* = −0.05 [*SE* = 0.12]; β = −0.03; *p* > 0.05) and GG (*B* = 0.13 [*SE* = 0.08]; β = 0.08; *p* > 0.05) on the dependent variable CY were not significant. Compared to the first regression, the relationship between the variables did not change noticeably after adding the covariates.

#### Reduced Professional Efficacy

Table A.4 highlights the results of two regressions with rPE as a dependent variable. Firstly, significant associations were found between the independent variable PM (*B* = −0.37 [*SE* = 0.09]; β = −0.37; *p* < 0.01), MM (*B* = −0.19 [*SE* = 0.08]; β = −0.19; *p* < 0.05), and rPE. A significant relationship between GG as an independent variable and rPE (*B* = −0.00 [*SE* = 0.06]; β = −0.01; *p* > 0.05) could not be identified. In the second model, after adding the six covariates, no striking changes in the regressions were found.

**Figure 1:**
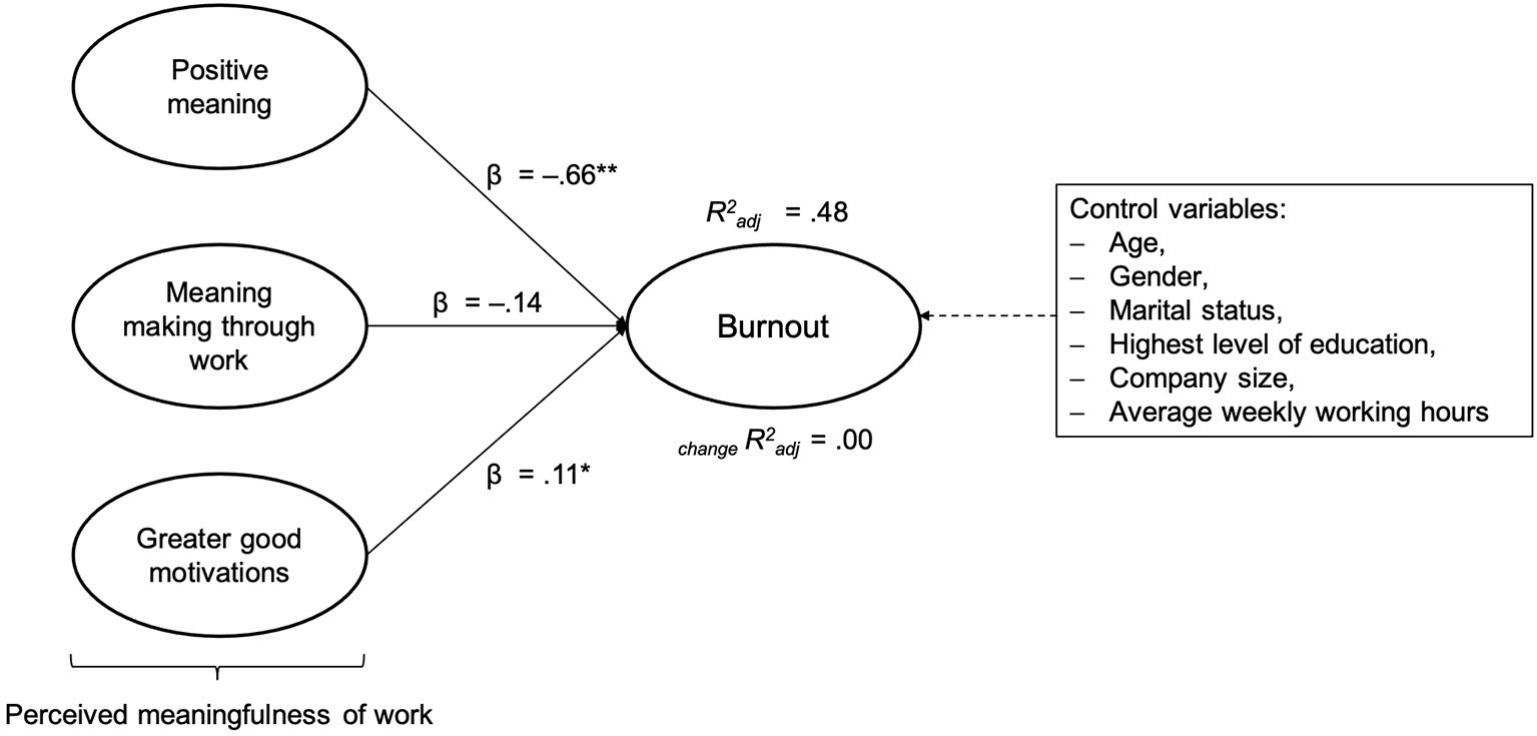
Multiple regression analysis with the total burnout score as a dependent variable and positive meaning (PM), meaning making through work (MM), and greater good motivations (GG) as independent variables (step 1); _change_$R_{\text{adj}}^{2}$ after incorporating the control variables (step 2). $R_{\text{adj}}^{2}$ = *R*^2^ adjusted; _change_$R_{\text{adj}}^{2}$ = change in *R*^2^ adjusted; *p < 0.05; **p < 0.01.

Approximately 48% of the variance of burnout, 23% of the variance of EE, 50% of the variance of CY, and 30% of the variance of rPE were explained by PM, MM, and GG. Furthermore, about 51% of the variance in work engagement can be accounted for by PM, MM, and GG. The results do not change markedly after controlling for the variables age, gender, highest level of education, company size, average weekly working hours, and marital status (see Tables A.1–A.5). As can be seen in Figure 1, the largest beta coefficient for burnout was the subscale PM (β = −0.66). This means that this variable made the strongest contribution toward explaining burnout.

Consequently, Hypothesis 1a was partly confirmed by the data analysis. A greater sense of perceived meaningfulness of work (two of the three subscales: PM and MM) is significantly negatively associated with burnout risk. Based on the multiple regression analyses, a negative relationship between GG and burnout could not be demonstrated (in contrast to the performed Pearson correlation, see Table 3). The weak positive association in the regression analysis could be explained by the assumption that the independent variable GG is associated with factors such as work pressure, stress, and low wellbeing (Moss, 2019). These factors are in turn related to burnout and the underlying burnout subscales (Maslach and Leiter, 2008; Yang, 2020; Lan et al., 2021). Furthermore, it is a conditional correlation because other covariates of perceived meaningfulness of work are included. This could mean that the other two subscales (i.e., PM and MM) are strong enough to reduce the singular correlation of GG; especially as the three subscales of perceived meaningfulness of work are strongly correlated with each other.

#### Work Engagement

As Table A.5 illustrates, our findings demonstrate significant associations between the independent variables PM (*B* = 0.73 [*SE* = 0.09]; β = 0.62; *p* < 0.01), MM (*B* = 0.25 [*SE* = 0.08]; β = 0.21; *p* < 0.01), and GG (*B* = −0.15 [*SE* = 0.06]; β = −0.14; *p* < 0.05) with work engagement. After adding the socio-demographic control variables, no noticeable changes could be identified in the strength, direction, and significance of the associations.

**Figure 2:**
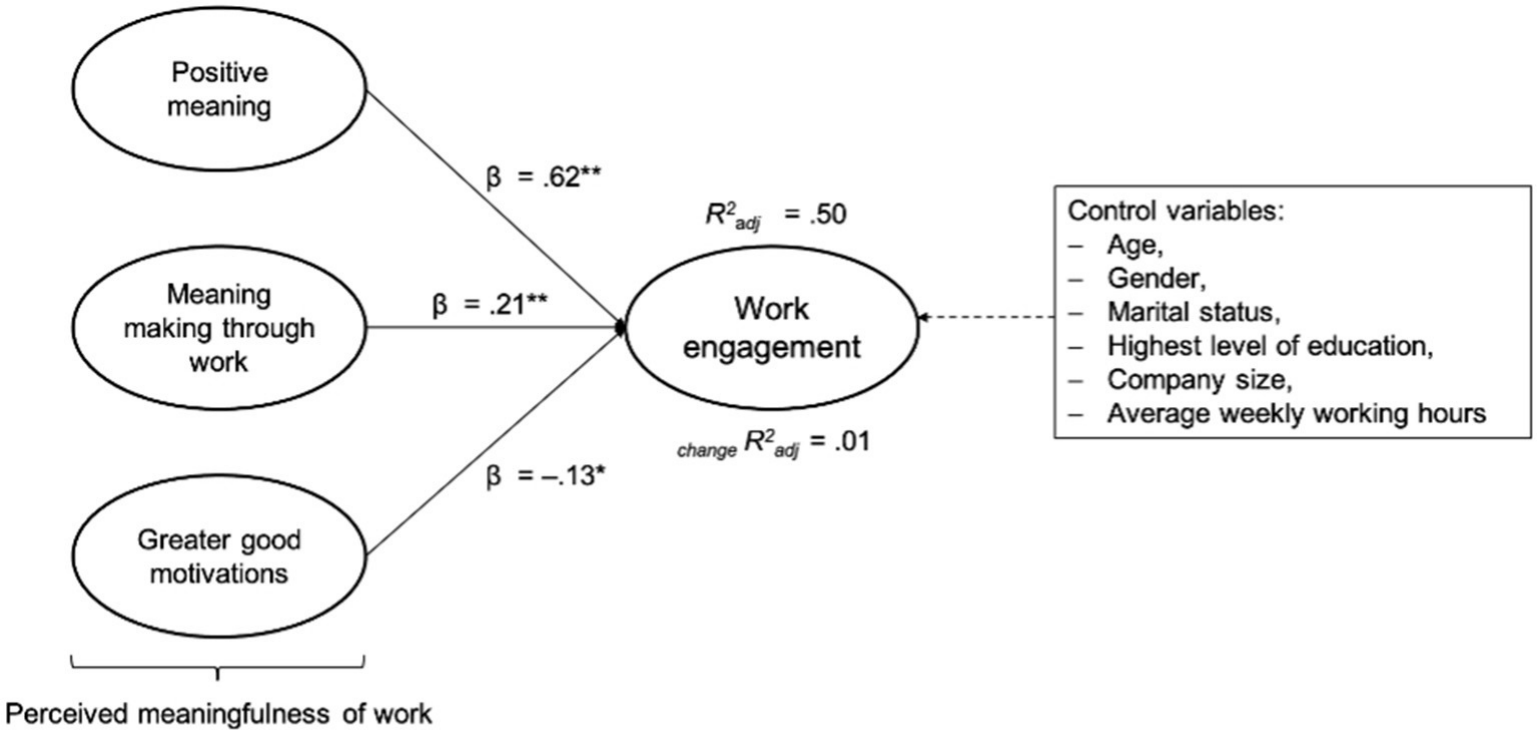
Multiple regression analysis with the total work engagement score as a dependent variable and PM, MM, and GG as independent variables (step 1); _change_$R_{\text{adj}}^{2}$ after incorporating the control variables (step 2). $R_{\text{adj}}^{2}$ = *R*^2^ adjusted; _change_$R_{\text{adj}}^{2}$ = change in *R*^2^ adjusted; *p < 0.05; **p < 0.01.

About 50% of the variance of work engagement was explained by PM, MM, and GG. The results do not change markedly after including the covariates (see Table A.5). As can be seen in Figure 2, the largest beta coefficient for work engagement was the subscale PM (β = 0.62). This means that this variable made the strongest contribution in explaining work engagement.

Thus, Hypothesis 1b was partly accepted based on the results. A greater sense of perceived meaningfulness of work (two of the three subscales: PM and MM) has a positive relationship with work engagement. Based on the multiple regression analyses, a positive relationship between GG and work engagement could not be demonstrated (in contrast to the performed Pearson correlation, see Table 3). The negative link in the regression analysis could be explained by considering that the independent variable GG is more likely to be the perception of having a positive impact on the common good (Harzer, 2016), which, however, is not accompanied by an actual subjective personal meaning (inherent psychological meaningfulness). Recent studies conceptualize work as a calling with the experience of perceived meaningfulness and not necessarily with the experience of meaningfulness and an organization (Steger and Dik, 2009; Geldenhuys et al., 2014) or as a way to have an impact on the GG. Another explanation might be that it is a conditional correlation. Consequently, it could be that the subscales PM and MM are sufficiently strong to reduce the association of GG.

### Hypotheses Testing for Perceived Sense of Homeliness and Burnout, and Perceived Sense of Homeliness and Work Engagement

Second, we tested the hypotheses that greater perceived sense of homeliness within a working environment is negatively associated with the risk of burnout (Hypothesis 2a) and positively associated with work engagement (Hypothesis 2b). The analyses follow the abovementioned procedure.

#### Burnout

Table A.6 shows the results of two regressions with burnout as a dependent variable. Firstly, we identified significant associations between burnout and the independent variables NF (*B* = −0.35 [*SE* = 0.06]; β = −0.34; *p* < 0.01), GM (*B* = −0.23 [*SE* = 0.07]; β = −0.23; *p* < 0.01), and EC (*B* = −0.25 [*SE* = 0.08]; β = −0.02; *p* < 0.01). A significant relationship between burnout and the independent variable IN (*B* = −0.02 [*SE* = 0.05]; β = −0.02; *p* > 0.05) could not be found. Secondly, after adding the aforementioned control variables, we found no obvious differences in comparison to the first model.

#### Emotional Exhaustion

Table A.7 highlights the results of two regressions with EE as a dependent variable. In the first step, significant relationships were found between EE and the independent variables NF (*B* = −0.52 [*SE* = 0.09]; β = −0.39; *p* < 0.01), and EC (*B* = −0.23 [*SE* = 11]; β = −0.15; *p* < 0.05). Significant relationships between EE and GM (*B* = −0.12 [*SE* = 0.09]; β = −0.09; *p* > 0.05) and IN (*B* = 0.08 [*SE* = 0.07]; β = 0.05; *p* > 0.05) could not be identified. In the second step, after adding the covariates, we found no noticeable changes.

#### Cynicism

The results of two regressions with the dependent variable CY are shown in Table A.8. Firstly, we demonstrated significant associations between the independent variables NF (*B* = −0.46 [*SE* = 0.10]; β = −0.30; *p* < 0.01), GM (*B* = −0.40 [*SE* = 0.12]; β = −0.26; *p* < 0.01), and EC (*B* = −0.37 [*SE* = 0.13]; β = −0.20; *p* < 0.01) on CY. A significant association between IN (*B* = 0.01 [*SE* = 0.08]; β = 0.01; *p* > 0.05) and CY was not identified. Secondly, after adding control variables, we found no striking differences compared to the first regression.

#### Reduced Professional Efficacy

Table A.9 shows the results of two regressions with rPE as a dependent variable. The first model demonstrates significant associations between the independent variables GM (*B* = −0.19 [*SE* = 0.06]; β = −0.21; *p* < 0.01), IN (*B* = −0.16 [*SE* = 0.05]; β = −0.16; *p* < 0.01), and EC (*B* = −0.16 [*SE* = 0.08]; β = −0.15; *p* < 0.05) on rPE. A significant relationship between the independent variable NF and rPE was not identified (*B* = −0.09 [*SE* = 0.06]; β = −0.10; *p* > 0.05). The second model, after adding the control variables, shows no noticeable differences in terms of significant associations between the independent variables GM and IN and rPE. As in the first model, there was no significant relationship between the independent variable NF and rPE. One noticeable change after adding the control variables was that the association between EC and rPE was no longer significant (*B* = −0.15 [*SE* = 0.09]; β = −0.14; *p* > 0.05).

**Figure 3:**
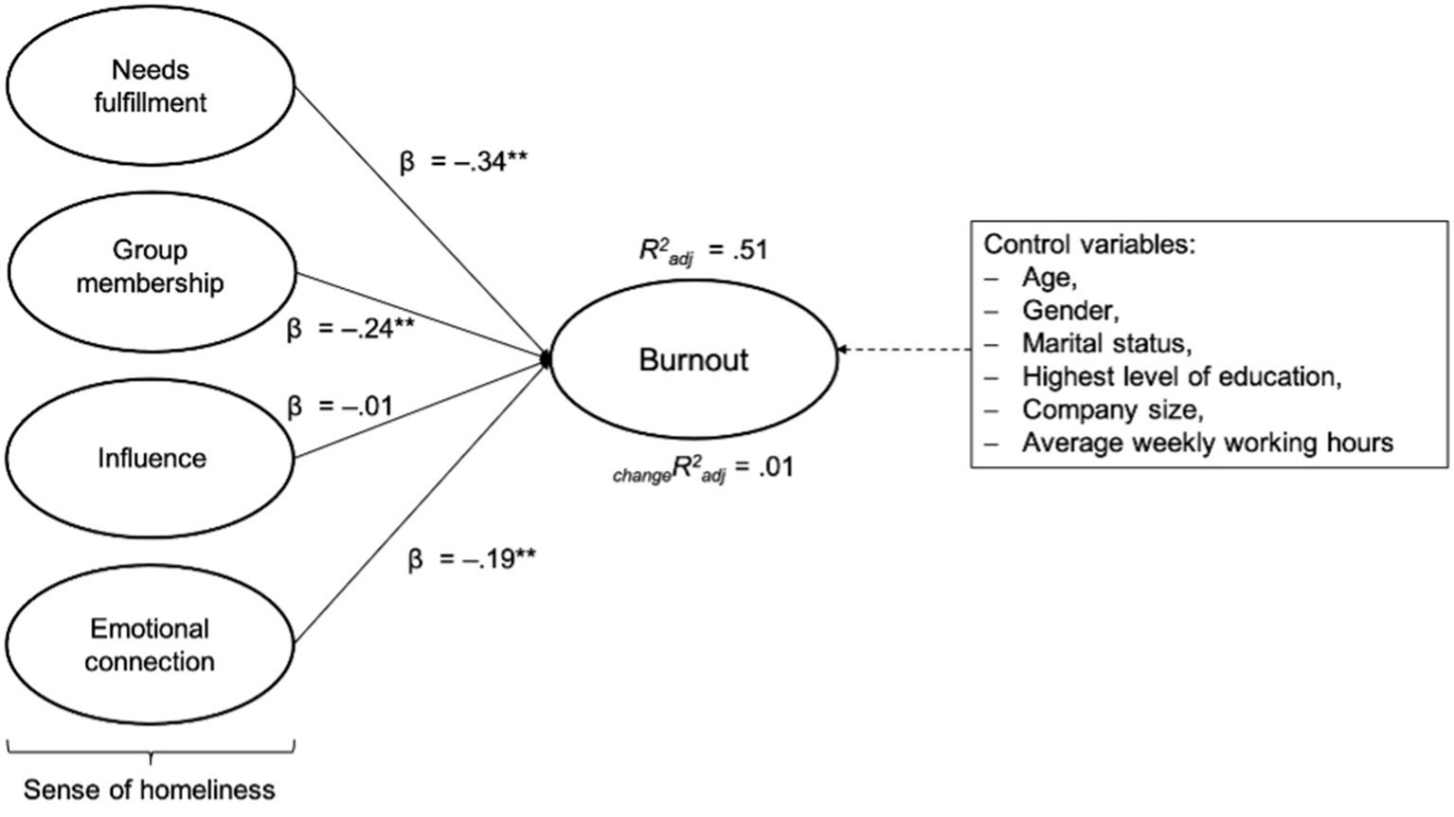
Multiple regression analysis with the total burnout score as a dependent variable and needs fulfillment (NF), group membership (GM), influence (IN), and emotional connection (EC) as independent variables (step 1); _change_$R_{\text{adj}}^{2}$ after incorporating the control variables (step 2). $R_{\text{adj}}^{2}$ = *R*^2^ adjusted; _change_$R_{\text{adj}}^{2}$ = change in *R*^2^ adjusted; *p < 0.05; **p < 0.01.

About 51% of the variance of burnout, 30% of the variance of EE, 48% of the variance of CY, and 29% of the variance of rPE were accounted for by NF, GM, IN, and EC. Similar to Hypothesis 1a, the results do not change substantially after controlling for age, gender, marital status, the highest attained educational level, company size, and average weekly working hours. As displayed in Figure 3, the largest beta coefficients for the total burnout score was NF (β = −0.34). This means that this variable made the strongest contribution in explaining burnout.

Thus, Hypothesis 2a is partly supported by the data analysis. A greater sense of homeliness of the working environment (three of the four subscales: NF, GM, and EC) is significantly negatively related with the risk of burnout. A significant relationship between the independent variable IN and the dependent variable burnout could not be found. IN here refers to the exercise of IN on the community with reciprocal IN of the community on oneself (Pretty et al., 2006). The current state of knowledge on the subscale IN within the construct sense of community is currently insufficient. However, our results suggest that the risk of burnout exists independently of the factor of IN.

#### Work Engagement

The results of two regressions with the dependent variable work engagement are shown in Table A.10. Here, the results of the first model demonstrate statistically significant associations between work engagement and the independent variables NF (*B* = 0.17 [*SE* = 0.06]; β = 0.19; *p* < 0.01), GM (*B* = 0.20 [*SE* = 0.07]; β = 0.06; *p* < 0.01), and EC (*B* = 0.45 [*SE* = 0.08]; β = 0.36; *p* < 0.01). Furthermore, no significant association could be found between IN and work engagement (*B* = 0.07 [*SE* = 0.05]; β = 0.06; *p* > 0.05). In the second model, after adding the covariates, we found no marked changes between the variables.

**Figure 4:**
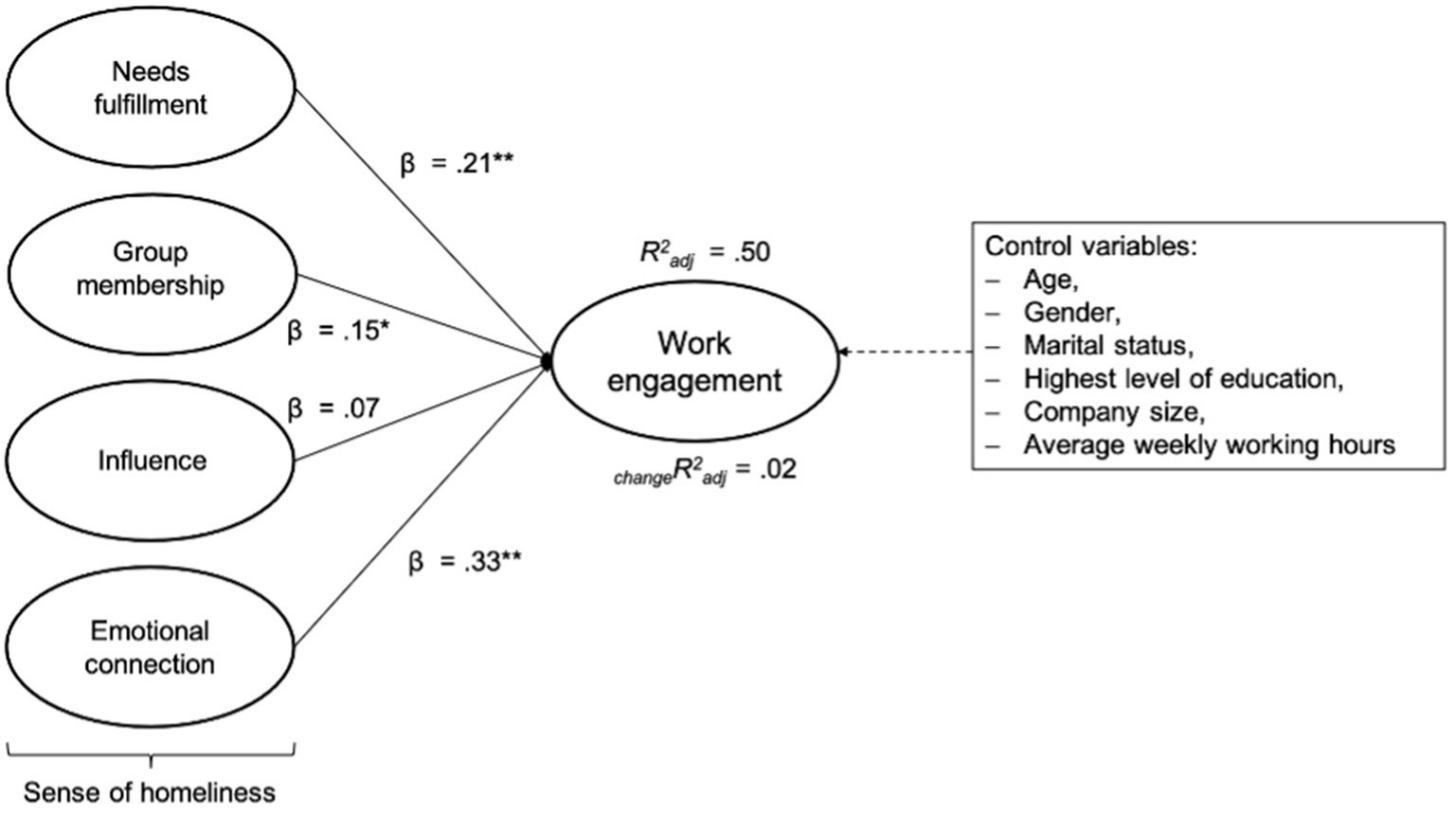
Multiple regression analysis with the total burnout score as a dependent variable and NF, GM, IN, and EC as independent variables (step 1); _change_$R_{\text{adj}}^{2}$ after incorporating the control variables (step 2) $R_{\text{adj}}^{2}$ = *R*^2^ adjusted; _change_$R_{\text{adj}}^{2}$ = change in *R*^2^ adjusted; ^*^p < 0.05; ^**^p < 0.01.

About 50% of the variance of work engagement were explained by NF, GM, IN, and EC. After controlling for age, gender, marital status, the highest attained level of education, company size, and average weekly working hours, the results do not change markedly (see Table A.10). As presented in Figure 4, the largest beta coefficient for work engagement was EC (β = 0.33). This means that this variable made the strongest individual contribution in explaining work engagement.

Consequently, Hypothesis 2b was partly confirmed by the results. A greater sense of homeliness (three of the four subscales: NF, GM, and EC) has a significant positive relationship with work engagement. Based on the multiple regression analysis, however, a significant positive relationship between IN and work engagement could not be demonstrated.

## Discussion

This is the first study to empirically examine the onset of burnout based on the bio-psycho-socio-spirito-cultural model postulated by Esch (2008a, 2011a, 2019) as the model of health and disease established by Engel (1977) does not seem to be sufficient to fully explain and describe the syndrome. Considering this extended model, the central aim of this study was to investigate the role of spirituality and work culture in the context of burnout, as well as work engagement (as it is commonly described as an antipode of burnout).

The results show that a greater sense of perceived meaningfulness of work is negatively associated with the risk of burnout (two of the three subscales: PM and MM), and positively associated with work engagement (two of the three subscales: PM and MM). Furthermore, we found that a greater sense of homeliness within the working environment (three of the four subscales: NF, GM, and EC) is negatively associated with the risk of burnout and positively associated with work engagement. Our preliminary results show that the established bio-psycho-social model is not sufficient to explain the onset of burnout on its own as the investigated factors cannot be clearly assigned to the established dimensions. For this reason, a paradigm shift (including two new, “semantic dimensions,” with spiritual and work cultural [sub-]dimensions) toward the “bio-psycho-socio-spirito-cultural model” seems necessary. Moreover, our results are consistent with and extend the findings of the few previous studies regarding meaningfulness of work (Fragoso et al., 2016; Van Wingerden and van der Stoep, 2017; Van Wingerden et al., 2018a,b), and sense of homeliness (Cicognani et al., 2009), in which similar moderate to high correlations were demonstrated. With respect to the studies on meaningfulness of work and burnout and meaningfulness of work and work engagement (Fragoso et al., 2016; Van Wingerden and van der Stoep, 2017; Van Wingerden et al., 2018a,b), we obtained more differentiated evidence regarding these relationships. In addition to total burnout, EE, and CY, we also considered rPE and found significant correlations between all variables. We also considered total perceived meaningfulness of work and all corresponding subscales of this construct. In addition, there have been very few studies so far on the relationship between sense of homeliness and burnout and sense of homeliness and work engagement (also considering the underlying subscales). With regard to the analyses on sense of homeliness and burnout (Cicognani et al., 2009), we also obtained more differentiated results for the subscales (for burnout and for sense of homeliness). In addition, we found evidence regarding the relationship between sense of homeliness and work engagement. The multiple regression analyses demonstrated that perceived meaningfulness of work and sense of homeliness of the working environment can explain a greater variance of CY and work engagement, than for EE or rPE.

The results of the multiple regression analyses also indicate that not all subscales of perceived meaningfulness of work and sense of homeliness are clearly associated with burnout and work engagement. According to the results, the subscales of PM, MM, and GG are significantly associated with burnout and work engagement. However, GG as a subscale of perceived meaningfulness of work do not seem to be clearly associated with burnout and work engagement. For sense of homeliness, the subscales of NF, GM, and EC are significantly associated with burnout and work engagement. Similarly, IN as a subscale of sense of homeliness is not significantly associated with burnout and work engagement. Although there is currently limited research regarding variables such as GG or IN, these results are consistent with other studies (e.g., Maslach and Leiter, 2008; Brown, 2012; Bagi, 2013; Adriaenssens et al., 2015), which show that burnout and reduced work engagement can also occur in professions that seem to have an impact on GG (e.g., physicians, nurses, and teachers), or that have IN within an organization/ a community (e.g., supervisors).

With regard to the socio-demographic variables (age, gender, highest level of education, company size, and weekly working hours), as in other studies (Maslach et al., 2001; Soares et al., 2007; Maslach and Leiter, 2008; Matin et al., 2012; Aguayo et al., 2017; Hakanen et al., 2019), few weak to medium or insignificant correlations could be identified. Only weak to medium correlations (see Table 4) were found between company size and perceived meaningfulness of work, as well as company size and sense of homeliness (except for NF). Other correlations were weak or not significant. Although the relationships between these variables are still largely unexplored, Hakanen et al. (2019) could not identify a significant correlation between company size and work engagement. Because the identified correlations for work engagement and the subscales are weak in our study, the significant result could be attributed to different assessments with respect to company size (Hakanen et al., 2019 recorded up to 500 employees and more while we measured up to 50,000 employees and more). Thus, the studies limited to companies with up to 500 employees might not be sufficient as associations regarding the mentioned variables would be expected for correspondingly larger companies. Nevertheless, our results in this domain are preliminary and had not been the focus of this study; they require further investigation.

Finally, the results of the multiple regression analyses, the corresponding variances, and the correlations of perceived meaningfulness of work, and sense of homeliness indicate that these are relevant factors associated with burnout and work engagement. We were able to provide evidence that perceived meaninfulness of work and sense of homeliness as psychological-mental or inner/interpersonal resources (understood as a feeling, experience, or sense) may also play a crucial role in the onset of burnout and work engagement. This finding corresponds with the assumption of Esch (2019), who describes burnout as a crisis of meaning and pleads for an extension of an understanding of health and disease based on a bio-psycho-socio-spirito-cultural model. Such an extended description (with two semantic dimensions, i.e., a spiritual and a work cultural dimension) of burnout, which is in line with our results and may demonstrate the need for coherence between the “inner world” (what is felt inside) and the “outer world” (the “being”), is thus considered fundamental (Esch, 2019).

### Sample Description

Comparing our sample with other investigations (e.g., Fragoso et al., 2016; Van Wingerden and van der Stoep, 2017; Van Wingerden et al., 2018a,b), we find few noticeable anomalies. In our data, the mean value (see Table 2) for burnout and the corresponding subscales are slightly higher (especially for EE; *M* = 4.20). This can be explained by the share of participants recruited in health service centers who were in psychiatric or psychotherapeutic treatment. A strong correlation between burnout and mental diseases such as depression, anxiety, or psychosomatic complaints has already been demonstrated by various authors (Melamed et al., 2006; Hapke et al., 2013; Koutsimani et al., 2019). For other (sub)scales, the data show no unexpected values.

Furthermore, as in previous studies (e.g., Fragoso et al., 2016; Van Wingerden and van der Stoep, 2017; Van Wingerden et al., 2018a,b) and following Ratner's (2009) interpretation of correlation coefficients, significant moderate to strong correlations (see Table 3) were found between perceived meaningfulness of work and burnout (*r* = −0.64) and perceived meaningfulness of work and work engagement (*r* = 0.65). A low correlation was only identified between EE and GG (*r* = −0.27). Thus, our study was able to replicate the results of previous studies and suggest new directions for future research of the syndrome.

### Implications

Firstly, based on the results of this study, we suggest that future studies further evaluate the relevance of the postulated bio-psycho-socio-spirito-cultural model, e.g., with reference to other psychological (e.g., depression, anxiety, and personality disorders), physical (e.g., pain and cardiovascular problems), and psychosomatic diseases (e.g., tinnitus, irritable bowel, and irritable bladder), and explore the role of stress-buffering resources such as spirituality (perceived meaningfulness, faith, and belief ≜ spirituality) and work culture (sense of homeliness, environment, belongingness, connectedness ≜ [work] culture).

Secondly, a correlation analysis with demographic factors showed that the company size (a higher number of employees) negatively correlates with the perceived meaningfulness of work, a sense of homeliness, and work engagement, and is positively related to burnout. As in several reviews of the literature (e.g., Alarcon, 2011; Adriaenssens et al., 2015; O'Connor et al., 2018), company size as a potential burnout-determining factor is missing entirely and, to date, the topic remains insufficiently investigated; future primary research could use both qualitative and quantitative methods to investigate the relationship between these mentioned factors. This could possibly provide important information on how to promote wellbeing and health, maintain productivity, and avoid sickness-related absences, fluctuation—and the associated loss of revenue.

**Figure 5:**
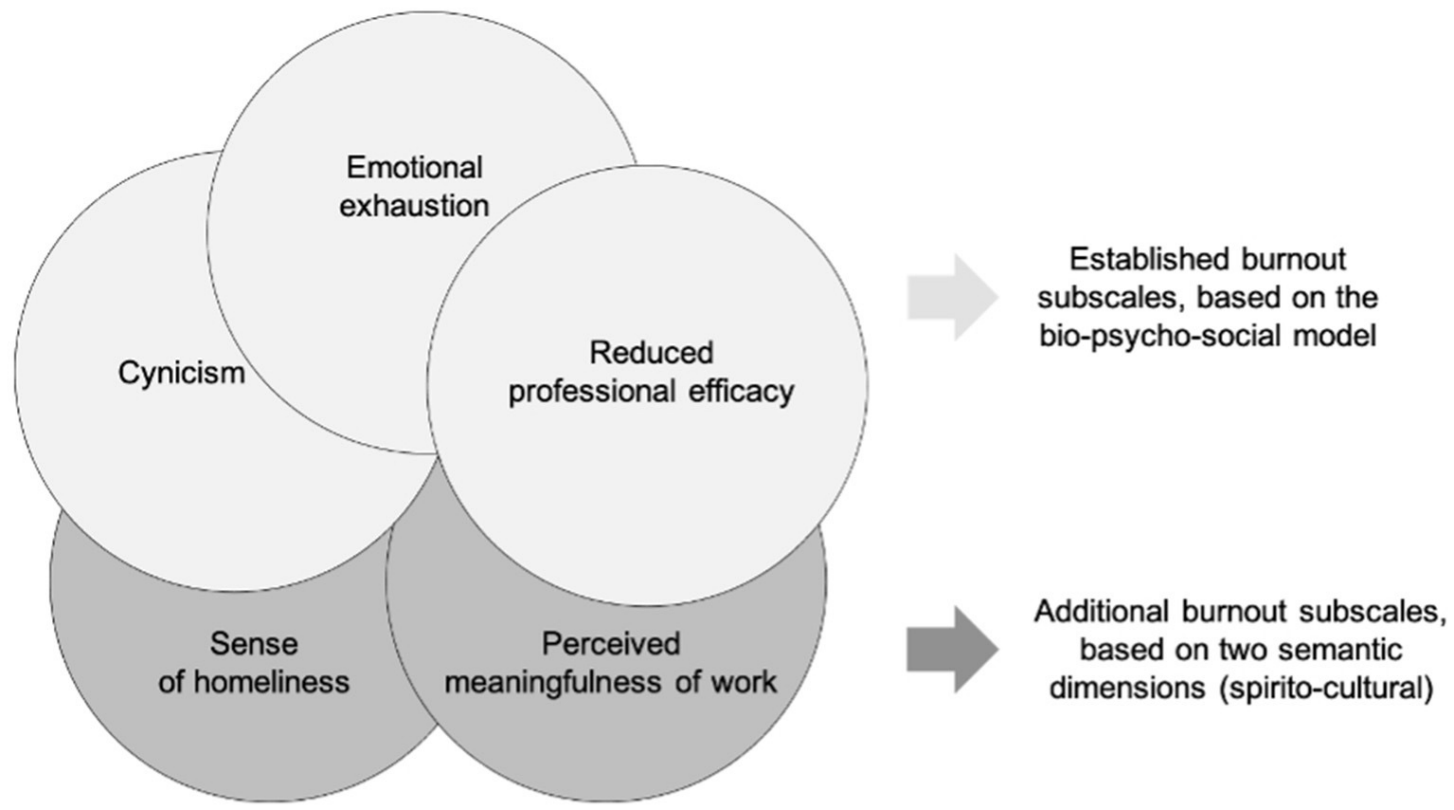
Extended concept of burnout. Own presentation, based on Esch (2019).

Thirdly, although this study was conducted using the MBI-GS, there is an open question as to whether this instrument with its underlying three burnout subscales is sufficient for describing burnout holistically and adequately, considering that the burnout concept was developed by Maslach et al. (1996) in the three-dimensional bio-psycho-social model. As has been suggested in initial studies, burnout is not only negatively related to work engagement (González-Romá et al., 2006), but also to the factors that can be attributed to a lack of perceived meaningfulness and a low sense of homeliness, community, or connectedness (Daniel, 2014; Van Wingerden and van der Stoep, 2017; Esch, 2019; Scanlan and Hazelton, 2019). For this reason, an extension of the current burnout concept (i.e., the inclusion of a spiritual and work cultural semantic dimension such as perceived meaningfulness and sense of homeliness and, thus, a correspondingly expanded approach to this syndrome), and measuring instrument, should be explored and discussed in further studies (see Figure 5).

Fourthly, besides theoretical implications, our findings also have practical implications for organizations, healthcare delivery, educational institutions, and for individuals in the work context. Hence, the most important practical implication of this study is that perceived meaningfulness of work (i.e., PM and MM) and sense of homeliness of the working environment (i.e., NF, GM, and EC) are important in the context of burnout and work engagement. With this insight, perceived meaningfulness of work and sense of homeliness could possibly become a key issue in companies. Thus, supervisors, human resource departments, and practitioners could be more aware of the role of perceived meaningfulness of work and sense of homeliness of the working environment and take appropriate actions. Based on perceived meaningfulness of work, (i) PM (work is perceived as important and meaningful) and (ii) MM (deepening an understanding of self and the world around and facilitating personal growth) should be encouraged. Exemplary interventions to promote these factors in a corporate context are appropriate objective agreements, which are based on corporate and employee values (employee and supervisor define targets based on selected corporate values that are in line with personal values), transparency (what are the targets and values of the company and the employee), and autonomy (delegate as many decisions as possible to the employees). Contrary to this, (iii) GG (a positive impact on the GG) (Steger et al., 2012) are not clearly associated with burnout and work engagement. Likewise, a sense of homeliness could be supported through measures that increase (i) NF (the perception that the needs of the employees are being fulfilled), (ii) GM (a sense of interpersonal relationships), and by (iii) EC (perceived solidarity, through shared history, location, or shared experience of the employees) (Peterson et al., 2008). This could be specifically promoted in a company context, for example, by considering personal and professional goals, communicating values, and celebrating achievements. In this context, in addition to regular target agreements, the measurement of these factors using quantitative (e.g., questionnaire) and qualitative methods (e.g., interviews through management or human resources) could be a possibility for identifying a lack of perceived meaningfulness of work and/or a sense of homeliness. Furthermore, these measures could be supplemented, for example, by individual discussions with supervisors or group activities.

Moreover, because burnout is a stress phenomenon, perceived meaningfulness and sense of homeliness should also be considered for other stress disorders. The consideration of these two additional factors could refer not only to the working context but also outside the working context (family, sports club, etc.). Therefore, perceived meaningfulness and sense of homeliness should be integrated into all phases of the therapy process [e.g., seven therapy phases within the self-management therapy by Kanfer et al. (2012)] or interventions and be sufficiently considered in clinical contexts. Integration into the various phases of therapy can be accompanied by the subjective, objective, assessment, and plan (SOAP) note, which provides a way for healthcare workers to document in a structured and an organized format (Cameron and Turtle-Song, 2002; Podder et al., 2020).

In this context, we also refer to mind-body medicine, which assumes that self-healing potentials can be activated *via* the interactions between brain, mind, body, and behavior. It includes behavioral approaches and techniques, which are used, for example, as part of a multimodal BERN stress management concept (Esch, 2020). The four columns of a professional and integrative stress management program are “behavior” (positive thinking and acting such as social interaction, social support, friendship, love, cognitive behavioral therapy, and motivational and positive psychology), “exercise” (aerobic and anaerobic physical activity), “relaxation” (including spirituality, faith, belief, meditation, mindfulness, and sleep hygiene), and “nutrition” (e.g., Mediterranean diet, including supplements—if indicated). The BERN concept aims to explore and reinforce health determinants and resistance resources (individual resilience and coherence factors) and reduce stress (e.g., Esch and Stefano, 2007, 2010; Esch, 2008a,b, 2020; Esch and Esch, 2016). Such a multimodal program could support people in dealing with stress and possibly strengthen psychological-mental or inner/interpersonal resources (such as spirituality, faith, belief, perceived meaningfulness, connectedness, and a sense of homeliness). The effect of such programs in dealing with burnout should be investigated both inside and outside the company.

Finally, considering the relationships between perceived meaningfulness of work and sense of homeliness on burnout and work engagement, as well as on overall life happiness (Van Wingerden and van der Stoep, 2017), appropriate interventions, such as the exploration of professional interests through career interest tests or stress management concepts, could take place within educational institutions (schools, universities, etc.). Such an intervention could highlight the importance of factors like PM and MM and to provide various career opportunities. The results of the present study demonstrate the need for potential training for employees to prevent health-threatening consequences.

### Limitations and Future Research

This study has some limitations. Firstly, even though we reached the calculated minimum sample size (340 participants) with 439 respondents, any cultural and regional differences that may have existed could not be analyzed. This implies a limited generalization of the results to other countries or cultures. Future studies should, therefore, take different regions or countries into consideration.

Secondly, all the used data were obtained through a self-reporting procedure, so that the possibility of bias due to response trends (e.g., social desirability, an error of extreme tendency, and social-desirability-response-set) cannot be entirely excluded. Future studies should expand a variety of methodologies, ideally implementing alternative and more objective methods to measure the variables and underlying concepts examined in this study (Podsakoff et al., 2003).

Thirdly, the cross-sectional nature does not allow for a deeper consideration of causality. To gain more information on the causal relationships between the variables, qualitative data collection and longitudinal studies are necessary. Qualitative studies could help to generate new hypotheses (Maudsley, 2011), whereas longitudinal studies with careful controls could allow exploring changes over time (VanderWeele et al., 2016).

Fourthly, we have cross-sectional and no panel data. We collected the cross-sectional data for the first time. There is no other cross and no panel data on this. Therefore, our study should be seen as a first milestone for research in this area. The collection of panel data is encouraged for upcoming studies.

Fifthly, more women (63.8%) participated in our study than men. According to Clerkin (2017), women attribute a greater importance to a meaningful work than men. This may have led not only to distortions in the context of perceived meaningfulness of work but also regarding sense of homeliness (e.g., when the development opportunities within the company are limited). Future studies should, therefore, try to obtain a more balanced sample.

Sixthly, the items of the WAMI and of BSCS may have been formulated too generally. This could have led to participants not having enough adequate response options if they felt, for example, emotionally exhausted or cynical for other reasons (e.g., effort–reward imbalance and value congruence). Future research should not only consider the semantic dimensions (containing the spiritual and work cultural dimensions) but also explore the concepts of sense of homeliness and perceived meaningfulness of work, in terms of their underlying subscales to provide differentiated information.

Seventhly, although there are currently no studies on the relationship between IN and work engagement, our results suggest that IN in the community may not be related to work engagement. One reason for this lack of relationship could be that IN refers to the interpersonal context, whereas work engagement refers to high level of energy and strong identification with one's work (no reference to colleagues, superiors). Thus, work engagement could be mainly related to internal aspects—independently of socially determined factors based on external IN and expectations, for example. Thus, further research regarding the interrelationships is needed.

Eighthly, based on an extended health and disease model, we followed two recruitment strategies to obtain a broad burnout score (i.e., low and high burnout levels) and also considered different industries. This increases the representativeness of our sample while it is still not representative for the whole German working population. There exists, to our knowledge, no representative data collection of employees, which includes the levels of spirituality or work culture. In any case, our study is the first to empirically investigate burnout based on the bio-psycho-socio-spirito-cultural model and should therefore offer suggestions for further research. Future studies should therefore attempt to generate a data set that is as representative as possible. Similarly, intervention studies should consider randomization to reduce the possibility of bias in the outcomes.

Finally, the present study examined the relationship between the variables in the work environment. However, the role of perceived meaningfulness and sense of homeliness outside the workplace (families, schools, etc.) and their relation to wellbeing and health in general remain open questions and should thus be explored further in future research. The investigation of burnout outside work (taking into account the semantic dimensions) seems necessary in the development and classification in the ICD-11 (burnout as an “occupational phenomenon”). Likewise, the examination and a holistic description of the syndrome (considering the underlying burnout subscales) are also important for the future Diagnostic and Statistical Manual of Mental Disorders (DSM-6).

## Conclusion

Burnout, which has been officially included in the ICD-11 by the WHO, will be attributed to chronic stress at work (WHO, 2021) from 2022. Based on the current bio-psycho-social model of health/disease, most burnout studies have concentrated on the investigation of biological, psychological, and social factors (e.g., Weber and Jaekel-Reinhard, 2000; Han, 2008; Seemüller et al., 2019). The use of the three-dimensional health and disease model may also be associated with an insufficient description of the burnout concept by Maslach et al. (1996). However, the other factors assigned to spirituality (e.g., perceived meaningfulness of work, belief, and faith) or work culture (e.g., sense of homeliness, connectedness, and belongingness) may also play an important role in stress and stress management (e.g., Esch and Stefano, 2007, 2010; Esch, 2008a, 2010, 2011a, 2019).

In summary, our results highlight the importance of an individual's/personal perception or “sense” that is linked to subjective feeling or experience, such as spirituality, and the perception or “sense” of homeliness, in the context of burnout and work engagement. Based on the results of this research, we were able to provide evidence that the currently used bio-psycho-social model is not sufficient to explain and describe the pathogenesis of burnout in a holistic and an encompassing way as spirituality and work culture are also related to burnout and work engagement. Overlooking core factors related to the onset and experience of burnout and work engagement, for example, can undermine description, diagnosis, prevention, and treatment and may lead to unnecessary suffering. Knowledge about the factors and effects of burnout should be constantly updated, revised, expanded, and challenged. If we do not reconsider the limits of our traditional models, concepts, and perspectives, we risk overlooking possible solutions—for people, organizations, economies, and society. For this reason, the expanded bio-psycho-socio-spirito-cultural model of health and disease, as postulated by Esch (2008a, 2011a, 2019), may be central to research and practice.

## Data Availability Statement

The original contributions presented in the study are included in the article/Supplementary Material, further inquiries can be directed to the corresponding authors.

## Ethics Statement

The studies involving human participants were reviewed and approved by University of Witten/Herdecke ethics committee (Germany; application no.: 76/2019). The patients/participants provided their written informed consent to participate in this study.

## Author Contributions

IL, TE, and MM designed the study. IL conducted the data collection, performed the data analysis, and drafted the manuscript. TE and MM reviewed and edited multiple versions of the manuscript, provided critical revisions, and acted as supervisors. All authors took part in result interpretation, read and approved the final manuscript.

## Conflict of Interest

The authors declare that the research was conducted in the absence of any commercial or financial relationships that could be construed as a potential conflict of interest.

## Publisher's Note

All claims expressed in this article are solely those of the authors and do not necessarily represent those of their affiliated organizations, or those of the publisher, the editors and the reviewers. Any product that may be evaluated in this article, or claim that may be made by its manufacturer, is not guaranteed or endorsed by the publisher.

## Footnotes

^1^We use the term “dimension” in the context of the (extended) model of health and disease.

^2^We use the term “factor” for variables related to burnout, stress, and wellbeing. Moreover, we abstain from using the term “determinant” to avoid causal interpretations that cannot be done based on cross-sectional data.

^3^The term “subscale” refers to the single components of a theoretical concept (e.g., burnout).

^4^Based on *t*-values and chi-squared tests, no differences in sociodemographic characteristics were found between the two samples (sample 1 online vs. sample 2 analogous), except for marital status (fewer individuals in sample 2 live in partnership), highest level of education (average level of education is lower in sample 2) and average weekly working hours (lower average weekly working hours in sample 2). Because individual group differences between the two populations seemed not to be relevant for testing the hypotheses, we decided to neglect them.

^5^For gender, the category of “other” (one participant) was not included in the point-biserial correlation.
