# Supplementary material for: An Empirical Investigation of the Relationship Between Spirituality, Work Culture, and Burnout: The Need for an Extended Health and Disease Model
Source: Front Psychol. 2021 Sep 13;12:723884. doi: 10.3389/fpsyg.2021.723884 (PMC8473891; doi:10.3389/fpsyg.2021.723884)
Supplement: Supplementary file 1 [file Table_1.pdf]

Supplementary Material

APPENDIX TABLE A.1 | Multiple Regression Analysis with Burnout as Dependent Variable and Positive Meaning, Meaning Making Through Work, and Greater Good Motivations as Independent Variables.

Model	Variables	Unstandardized coefficient		Standardized coefficient	p	R^2_{adj}	F
		B	SE	β			
1	(Constant)	6.18	.16	–	.00**	.48	134.21 (3; 435)
	Positive meaning	–.76	.09	–.65	.00**		
	Meaning making through work	–.16	.08	–.14	.05*		
	Greater good motivations	.12	.06	.10	.04*		
2	(Constant)	6.63	.64	–	.00**	.48	18.70 (23; 415)
	Positive meaning	–.77	.10	–.66	.00**		
	Meaning making through work	–.16	.09	–.14	.06		
	Greater good motivations	.12	.06	.11	.05*		

Model 1: First regression, associations between burnout and positive meaning, meaning making through work, and greater good motivations.
Model 2: Second regression, associations after adding the covariates (age, gender, highest level of education, company size, average weekly working hours, and marital status).
*p < .05. **p < 0.01. – statistically significant (two-tailed), results are based on 1000 bootstrap samples.

Extended Health and Disease Model

TABLE A.2 | Multiple Regression Analysis with Emotional Exhaustion as Dependent Variable and Positive Meaning, Meaning Making Through Work, and Greater Good Motivations as Independent Variables.

Model	Variables	Unstandardized coefficient		Standardized coefficient	p	R^2_{adj}	F
		B	***SE***	**β**			
1	(Constant)	6.56	.23	–	.00**	.22	41.69 (3; 435)
	Positive meaning	–.64	.14	–.44	.00**		
	Meaning making through work	–.24	.12	–.16	.06		
	Greater good motivations	.22	.09	.16	.02*		
2	(Constant)	8.31	1.28	–	.00**	.23	6.53 (23; 415)
	Positive meaning	–.68	.15	–.46	.00**		
	Meaning making through work	–.26	.12	–.18	.04*		
	Greater good motivations	.24	.10	.17	.01*		

Model 1: First regression, associations between emotional exhaustion and positive meaning, meaning making through work, and greater good motivations.
Model 2: Second regression, associations after adding the covariates (age, gender, highest level of education, company size, average weekly working hours, and marital status).
**p* < .05. ***p* < 0.01. – statistically significant (two-tailed), results are based on 1000 bootstrap samples.

Extended Health and Disease Model

TABLE A.3 | Multiple Regression Analysis with Cynicism as Dependent Variable and Positive Meaning, Meaning Making Through Work, and Greater Good Motivations as Independent Variables.

Model	Variables	Unstandardized coefficient		Standardized coefficient	*p*	R^2_{adj}	*F*
		B	***SE***	**β**			
1	(Constant)	7.60	.23	–	.00**	.50	146.48 (3; 435)
	Positive meaning	–1.27	.12	–.74	.00**		
	Meaning making through work	–.05	.12	–.03	.68		
	Greater good motivations	.13	.08	.08	.11		
2	(Constant)	7.63	.72	–	.00**	.50	20.26 (23; 415)
	Positive meaning	–1.23	.12	–.72	.00**		
	Meaning making through work	–.06	.12	–.03	.61		
	Greater good motivations	.11	.08	.07	.19		

Model 1: First regression, associations between cynicism and positive meaning, meaning making through work, and greater good motivations.
Model 2: Second regression, associations after adding the covariates (age, gender, highest level of education, company size, average weekly working hours, and marital status).
**p* < .05. ***p* < 0.01. – statistically significant (two-tailed), results are based on 1000 bootstrap samples.

Extended Health and Disease Model

TABLE A.4 | Multiple Regression Analysis with Reduced Professional Efficacy as Dependent Variable and Positive Meaning, Meaning Making Through Work, and Greater Good Motivations as Independent Variables.

Model	Variables	Unstandardized coefficient		Standardized coefficient	p	R^2_{adj}	F
		B	SE	β			
1	(Constant)	4.36	.18	–	.00**	.29	60.90 (3; 435)
	Positive meaning	–.37	.09	–.37	.00**		
	Meaning making through work	–.19	.08	–.19	.01*		
	Greater good motivations	–.00	.06	–.01	.94		
2	(Constant)	3.96	.30	–	.00**	.30	9.26 (23; 415)
	Positive meaning	–.41	.09	–.41	.00**		
	Meaning making through work	–.17	.08	–.17	.03*		
	Greater good motivations	.01	.06	.02	.82		

Model 1: First regression, associations between reduced professional efficacy and positive meaning, meaning making through work, and greater good motivations.
Model 2: Second regression, associations after adding the covariates (age, gender, highest level of education, company size, average weekly working hours, and marital status).
*p < .05. **p < 0.01. – statistically significant (two-tailed), results are based on 1000 bootstrap samples.

Extended Health and Disease Model

TABLE A.5 | Multiple Regression Analysis with Work Engagement as Dependent Variable and Positive Meaning, Meaning Making Through Work, and Greater Good Motivations as Independent Variables.

Model	Variables	Unstandardized coefficient		Standardized coefficient	*p*	R^2_{adj}	*F*
		B	*SE*	β			
1	(Constant)	1.65	.16	–	.00**	.50	146.09 (3; 435)
	Positive meaning	.73	.09	.62	.00**		
	Meaning making through work	.25	.08	.21	.00**		
	Greater good motivations	–.15	.06	–.14	.01*		
2	(Constant)	1.97	.46	–	.00**	.51	20.64 (23; 415)
	Positive meaning	.73	.10	.62	.00**		
	Meaning making through work	.25	.09	.21	.01**		
	Greater good motivations	–.15	.06	–.13	.01*		

Model 1: First regression, associations between work engagement and positive meaning, meaning making through work, and greater good motivations.
Model 2: Second regression, associations after adding the covariates (age, gender, highest level of education, company size, average weekly working hours, and marital status).
*$p < .05$. **$p < 0.01$. – statistically significant (two-tailed), results are based on 1000 bootstrap samples.

Extended Health and Disease Model

TABLE A.6 | Multiple Regression Analysis with Burnout as Dependent Variable and Needs Fulfillment, Group Membership, Influence, and Emotional Connection as Independent Variables.

Model	Variables	Unstandardized coefficient		Standardized coefficient	*p*	R^2_{adj}	*F*
		B	***SE***	**β**			
1	(Constant)	6.40	.21	–	.00**	.50	111.14 (4; 434)
	Needs fulfillment	–.35	.06	–.34	.00**		
	Group membership	–.23	.07	–.23	.00**		
	Influence	–.02	.05	–.02	.67		
	Emotional connection	–.25	.08	–.20	.00**		
2	(Constant)	6.91	.69	–	.00**	.51	19.62 (4; 414)
	Needs fulfillment	–.36	.06	–.34	.00**		
	Group membership	–.24	.07	–.24	.00**		
	Influence	–.01	.06	–.01	.83		
	Emotional connection	–.23	.09	–.19	.00**		

Model 1: First regression, associations between burnout and needs fulfillment, group membership, influence, and emotional connection.
Model 2: Second regression, associations after adding the covariates (age, gender, highest level of education, company size, average weekly working hours, and marital status).
*p < .05. **p < 0.01. – statistically significant (two-tailed), results are based on 1000 bootstrap samples.

Extended Health and Disease Model

TABLE A.7 | Multiple Regression Analysis with Emotional Exhaustion as Dependent Variable and Needs Fulfillment, Group Membership, Influence, and Emotional Connection as Independent Variables.

Model	Variables	Unstandardized coefficient		Standardized coefficient	p	R^2_{adj}	F
		B	SE	β			
1	(Constant)	7.00	.27	–	.00**	.30	47.53 (4; 434)
	Needs fulfillment	–.52	.09	–.39	.00**		
	Group membership	–.12	.09	–.09	.19		
	Influence	.08	.07	.05	.28		
	Emotional connection	–.23	.11	–.15	.04*		
2	(Constant)	8.76	1.10	–	.00**	.30	8.73 (24; 414)
	Needs fulfillment	–.50	.09	–.38	.00**		
	Group membership	–.17	.10	–.13	.08		
	Influence	.08	.08	.06	.34		
	Emotional connection	–.22	.12	–.14	.06		

Model 1: First regression, associations between emotional exhaustion and needs fulfillment, group membership, influence, and emotional connection.
Model 2: Second regression, associations after adding the covariates (age, gender, highest level of education, company size, average weekly working hours, and marital status).
*p < .05. **p < 0.01. – statistically significant (two-tailed), results are based on 1000 bootstrap samples.

Extended Health and Disease Model

TABLE A.8 | Multiple Regression Analysis with Cynicism as Dependent Variable and Needs Fulfillment, Group Membership, Influence, and Emotional Connection as Independent Variables.

Model	Variables	Unstandardized coefficient		Standardized coefficient	*p*	R^2_{adj}	*F*
		B	***SE***	**β**			
1	(Constant)	7.69	.30	–	.00**	.47	97.84 (4; 434)
	Needs fulfillment	–.46	.10	–.30	.00**		
	Group membership	–.40	.12	–.26	.00**		
	Influence	.01	.08	.01	.88		
	Emotional connection	–.37	.13	–.20	.00**		
2	(Constant)	7.78	.68	–	.00**	.48	18.04 (24; 414)
	Needs fulfillment	–.50	.11	–.32	.00**		
	Group membership	–.37	.12	–.24	.00**		
	Influence	.04	.09	.02	.69		
	Emotional connection	–.33	.13	–.18	.02*		

Model 1: First regression, associations between cynicism and needs fulfillment, group membership, influence, and emotional connection.
Model 2: Second regression, associations after adding the covariates (age, gender, highest level of education, company size, average weekly working hours, and marital status).
**p* < .05. ***p* < 0.01. – statistically significant (two-tailed), results are based on 1000 bootstrap samples.

Extended Health and Disease Model

TABLE A.9 | Multiple Regression Analysis with Reduced Professional Efficacy as Dependent Variable and Needs Fulfillment, Group Membership, Influence, and Emotional Connection as Independent Variables.

Model	Variables	Unstandardized coefficient		Standardized coefficient	*p*	R^2_{adj}	*F*
		B	*SE*	β			
1	(Constant)	4.50	.20	–	.00**	.28	44.18 (4; 434)
	Needs fulfillment	–.09	.06	–.10	.13		
	Group membership	–.19	.06	–.21	.00**		
	Influence	–.16	.05	–.16	.00**		
	Emotional connection	–.16	.08	–.15	.04*		
2	(Constant)	4.20	.40	–	.00**	.29	8.52 (24; 414)
	Needs fulfillment	–.08	.06	–.09	.24		
	Group membership	–.20	.07	–.22	.00**		
	Influence	–.16	.05	–.17	.00**		
	Emotional connection	–.15	.09	–.14	.09		

Model 1: First regression, associations between reduced professional efficacy and needs fulfillment, group membership, influence, and emotional connection.
Model 2: Second regression, associations after adding the covariates.
*$p < .05$. **$p < 0.01$. – statistically significant (two-tailed), results are based on 1000 bootstrap samples.

Extended Health and Disease Model

TABLE A.10 | Multiple Regression Analysis with Work Engagement as Dependent Variable and Needs Fulfillment, Group Membership, Influence, and Emotional Connection as Independent Variables.

Model	Variables	Unstandardized coefficient		Standardized coefficient	*p*	R^2_{adj}	*F*
		B	*SE*	β			
1	(Constant)	1.35	.21	–	.00**	.48	100.33 (4; 434)
	Needs fulfillment	.17	.06	.16	.00**		
	Group membership	.20	.07	.19	.00**		
	Influence	.07	.05	.06	.20		
	Emotional connection	.45	.08	.36	.00**		
2	(Constant)	1.60	.54	–	.00**	.50	18.86 (24; 414)
	Needs fulfillment	.22	.06	.21	.00**		
	Group membership	.16	.07	.15	.04*		
	Influence	.08	.06	.07	.15		
	Emotional connection	.41	.08	.33	.00**		

Model 1: First regression, associations between work engagement and needs fulfillment, group membership, influence, and emotional connection.
Model 2: Second regression, associations after adding the covariates (age, gender, highest level of education, company size, average weekly working hours, and marital status).
*p < .05. **p < 0.01. – statistically significant (two-tailed), results are based on 1000 bootstrap samples.
